# Engineering edgeless human skin with enhanced biomechanical properties

**DOI:** 10.1126/sciadv.ade2514

**Published:** 2023-01-27

**Authors:** Alberto Pappalardo, David Alvarez Cespedes, Shuyang Fang, Abigail R. Herschman, Eun Young Jeon, Kristin M. Myers, Jeffrey W. Kysar, Hasan Erbil Abaci

**Affiliations:** ^1^ Department of Dermatology, Columbia University Irving Medical Center, New York, NY 10032, USA.; ^2^ Department of Mechanical Engineering, School of Engineering and Applied Science, Columbia University, New York, NY 10027, USA.; ^3^ Department of Otolaryngology - Head & Neck Surgery, Columbia University Irving Medical Center, New York, NY 10032, USA.

## Abstract

Despite the advancements in skin bioengineering, 3D skin constructs are still produced as flat tissues with open edges, disregarding the fully enclosed geometry of human skin. Therefore, they do not effectively cover anatomically complex body sites, e.g., hands. Here, we challenge the prevailing paradigm by engineering the skin as a fully enclosed 3D tissue that can be shaped after a body part and seamlessly transplanted as a biological clothing. Our wearable edgeless skin constructs (WESCs) show enhanced dermal extracellular matrix (ECM) deposition and mechanical properties compared to conventional constructs. WESCs display region-specific cell/ECM alignment, as well as physiologic anisotropic mechanical properties. WESCs replace the skin in full-thickness wounds of challenging body sites (e.g., mouse hindlimbs) with minimal suturing and shorter surgery time. This study provides a compelling technology that may substantially improve wound care and suggests that the recapitulation of the tissue macroanatomy can lead to enhanced biological function.

## Teaser

Human skin is bioengineered as a wearable tissue with anatomical shapes tailored to perfectly match recipient sites.

## INTRODUCTION

The human skin is a complex organ to bioengineer and repair because of its diverse cellular makeup, unique anatomy, and body site–specific cellular and mechanical properties. Recreating this complexity in vitro using human cells has notable implications on personalized skin replacement therapy and human-relevant skin disease modeling and drug screening. In the past decade, there has been major progress toward recapitulating the cellular diversity of the skin including our and others’ recent efforts on incorporating different skin components, e.g., microvasculature, hair follicles, and immune cells, into three-dimensional (3D) skin constructs, getting us closer to the real human skin (*1*–*18*).

Despite these advancements, the current method that we use today to reconstruct skin cells in 3D still follows the same approach that was first introduced nearly 40 years ago (*19*). The current paradigm contemplates 3D skin constructs as rectangular or circular planar patches with open boundaries on all sides and disregards the fact that human skin is a fully enclosed organ and has complex geometries. While the conventional skin constructs can be successfully grafted onto flat body parts, such as the upper back (*20*), they typically fail to effectively cover irregular body parts, such as fingers or facial features, because of their generic geometry and limited mechanical properties (*21*). They have to be delicately placed as multiple patches (e.g., around each finger) on large wounds, requiring a high number of stitches in between each individual piece or excessive bandaging, substantially lengthening the surgery time and worsening the aesthetic and functional outcome of the procedure.

The human skin is a continuous, fully enclosed organ, where biophysical forces between cells and the surrounding extracellular matrix (ECM) are in a mechanical balance at homeostasis (*22*). In addition, it has regional structural variations contributing to body site–specific responses during skin regeneration and wound healing (*23*, *24*). The 3D skin constructs generated using the current approach do not truly reflect the homeostatic biomechanical environment of the healthy human skin or capture region-specific structural differences due in part to their discontinuous boundaries and generic geometries.

In this study, we challenge the prevailing paradigm in skin bioengineering by reimagining 3D skin constructs as fully enclosed continuous 3D tissues that can be seamlessly transplanted as a biological clothing (e.g., skin gloves) on any part of the body. We hypothesized that 3D skin constructs built as a continuous tissue would recreate the biophysical interactions and cellular/extracellular organization found in the human skin in a body site–specific fashion, leading to superior mechanical and functional properties compared to conventional hydrogel-based skin constructs. To this end, we leveraged the capabilities of the Carbon Digital Light Synthesis technology (DLS) to tackle the challenge of making fully enclosed 3D skin in various shapes with a cornified epidermis achieved through an air-liquid interface (ALI) culture under continuous medium perfusion. Our technology provides full-thickness human skin constructs with enhanced extracellular and mechanical properties, geometry-dependent cellular/extracellular responses, integrated perfusion capabilities, and personalized wearable shapes for precision skin replacement therapy.

## RESULTS

### Establishing the method to engineer wearable edgeless human skin

Our first aim was to develop a method that can reproducibly generate 3D human skin as a fully enclosed and continuous tissue in custom shapes. As one of the geometries of interests in this study, we used the human hand because of its anatomical complexity and potential downstream clinical applications, such as hand surgery, by envisioning that the 3D skin can be transplanted as a single-piece wearable tissue, e.g., skin glove, customized for the patient, minimizing the need for suturing and substantially reducing the length of surgeries.

**Fig. 1.**
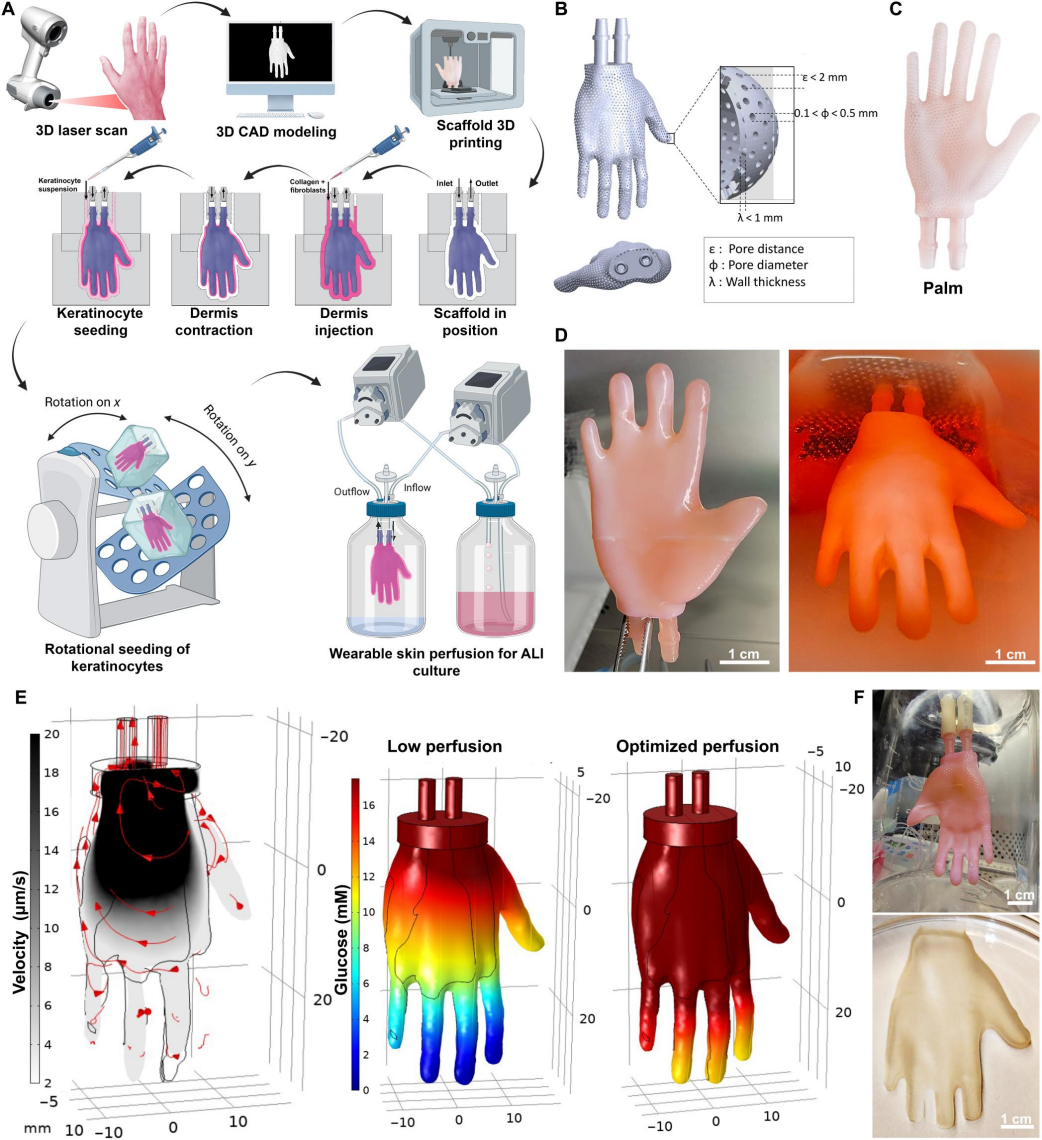
Development of the WESCs. (**A**) Pipeline of the WESC fabrication: A CAD model of an anatomical region is generated through laser scanning and used as a template to design and 3D-print a hollow, perfusable, and porous scaffold through DLS printing. The dermis is casted on the scaffold using a PDMS negative mold. After 14 days of submerging in culture, the dermis contracts and remodels; at this time, KCs are seeded on the surface of the dermis, injecting them as a single-cell suspension on the offset between the dermis and the PDMS mold. A rocking platform ensures that the KCs attach to the dermis surface evenly. After up to 7 days of additional submerging in culture, the scaffold is connected to the fluidic system, and the WESC is cultured inside a glass bottle at the ALI. (**B**) CAD model of the hand scaffold with technical specifics. (**C**) Photograph of a 3D-printed hand scaffold. (**D**) Hand-shaped dermis after 14 days of remodeling. (**E**) COMSOL simulation of the velocity profile (left; arrows indicate the direction of the flow) and the glucose concentration throughout the scaffold under low (middle) and optimized perfusion conditions (right). (**F**) WESC on day 7 of ALI culture (up) and after being removed from the scaffold as a skin glove. Scale bars, 1 cm (D and F). Part of the illustration (A) was designed with Biorender.

Our protocol starts by using a 3D laser scanner to generate a computer-aided design (CAD) model of a body part, e.g., human hand (Fig. 1A). On the basis of the geometrical features of the CAD model, we design a hollow scaffold (e.g., skin scaffold) with a permeable porous wall (pore distance of <2 mm and diameter of <0.5 mm) and an inlet and outlet port for medium perfusion and ALI culture (Fig. 1B and fig. S1). After 3D printing the scaffold (Fig. 1C) using the Carbon DLS method, we cast the dermis [a suspension of human primary fibroblasts (FBs) and collagen I] around the exterior surface of the scaffold using a polydimethylsiloxane (PDMS) negative mold and culture the dermal construct submerged in the medium for 14 days to allow for ECM remodeling (Fig. 1D and fig. S2). Then, we inject in the space between the dermal construct and the mold human primary keratinocytes (KCs) resuspended in culture medium. To achieve an even distribution of KCs, we place the assembly on an orbital rocking platform in a cell culture incubator for 4 hours, and then after KC attachment, we remove the skin construct from the mold and submerge it in epidermalization (EPI) medium for up to 7 days. To bring the construct to ALI culture, which is required for proper cornification of the skin, we connect the inlet and outlet ports of the scaffold to external tubing, suspend the construct in a glass bottle exposing it to air, and perfuse the construct through the scaffold with cornification (CORN) medium using a peristaltic pump. Throughout this process, the tissue is always supported by the inner 3D-printed scaffold on which it wraps around, while the external PDMS molds are removed after each specific phase that they are involved with (dermis casting and KC seeding). We ran in silico COMSOL simulations to determine the minimum perfusion rate needed to achieve an adequate level of convectional transport yielding a sufficient glucose concentration throughout the construct (Fig. 1E and fig. S3). After up to 7 days of ALI culture at the optimized perfusion rate (e.g., 5 ml/min for the hand design), the wearable edgeless skin construct (WESCs) are ready for downstream in vitro applications, such as drug testing, or can be explanted from the scaffold as a single piece (Fig. 1F and movie S1) for in vivo transplantation.

**Fig. 2.**
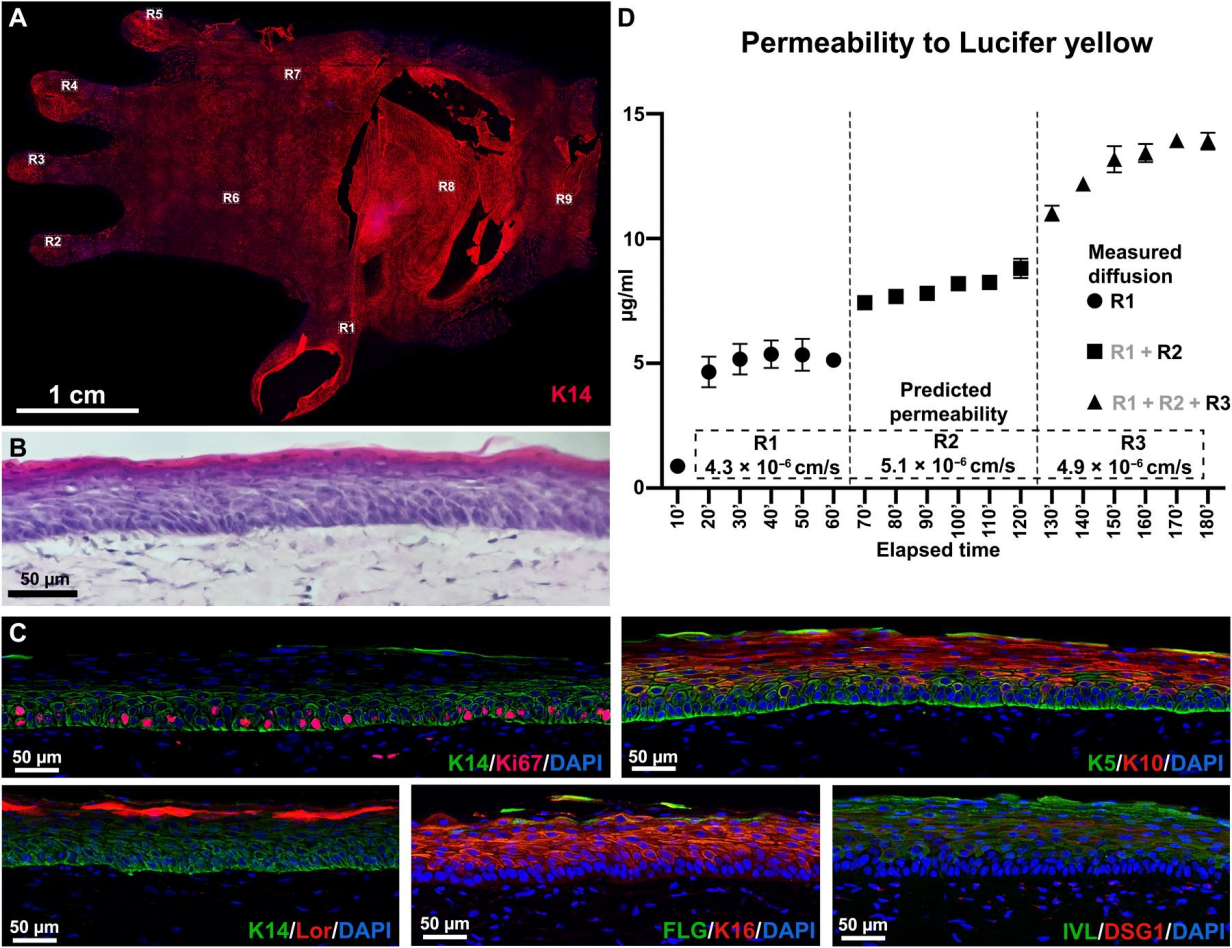
Validation of the epidermis in the WESCs. (**A**) Mapping of KC coverage on the palmar surface of the skin glove using Keratin 14 IF staining; the whole palmar surface of the skin glove was stained for Keratin 14, highly expressed in basal KCs, and scanned to demonstrate a full epidermal coverage. (**B**) H&E staining of WESC sections showing the epidermis and dermis compartments. (**C**) IF images demonstrating the physiological expression of several key epidermal markers. The panel confirms the proper development of all specific layers of the epidermis with Keratin 14 (K10), Keratin 5 (K5), and Ki67 in the basal layer; Keratin 10 (K10), Keratin 16 (K16), Desmoglein 1 (DSG1), and involucrin (IVL) in the stratum spinosum; Keratin 10, involucrin, filaggrin, and loricrin (Lor) in the stratum granulosum; and filaggrin and loricrin in the stratum corneum. (**D**) Graph showing the progressively increasing Lucifer yellow concentration in the medium reservoir; two additional regions, R2 and R3, were tested sequentially after 60 and 120 min, respectively, and the samples were collected every 10 min for a total of 180 min. The epidermal permeabilities of the regions were numerically estimated on the basis of the experimental data. Scale bars, 1 cm (A) and 50 μm (B and C). DAPI, 4′,6-diamidino-2-phenylindole.

To confirm the full coverage of the epidermis in the skin glove, we performed immunofluorescence (IF) staining of Keratin 14 and scanned the entire palmar surface (Fig. 2A). We further scanned at higher magnification nine representative regions including the wrist, palm, and all fingers to highlight the details at single-cell resolution (fig. S4). To further demonstrate the proper formation and maturation of the epidermis on WESCs, we performed hematoxylin and eosin (H&E) (Fig. 2B) and IF staining (Fig. 2C) to detect all four epidermal layers: a proliferative stratum basale (Keratin 14, Keratin 5, and Ki67), the stratum spinosum (Keratin 10, involucrin, and desmoglein 1), the stratum granulosum (Keratin 10, filaggrin, and loricrin), and the stratum corneum (filaggrin and loricrin). To functionally confirm the uniform epidermal coverage and the barrier function, we measured the permeability of a fluorescent dye, Lucifer yellow, at multiple regions that have an area of 1 cm^2^ (fig. S5). The dye concentration in the circulating medium increased constantly during the 3 hours with a linear trend (Fig. 2D). The in silico simulations plugged with our time-lapse data estimated that all three regions have similar epidermal permeabilities, suggesting the formation of a uniform epidermis throughout the WESCs.

### Enhanced dermis formation in wearable edgeless skin

We postulated that the fully enclosed and continuous geometry of the WESCs would recapitulate the biophysical interactions and cellular/extracellular organization of the human dermis in a body site–specific fashion, leading to superior mechanical and functional properties compared to conventional skin constructs. To corroborate our hypothesis, we first generated and used a dermis-only WESC model with a simplified cylindrical geometry (figs. S1 and S2).

**Fig. 3.**
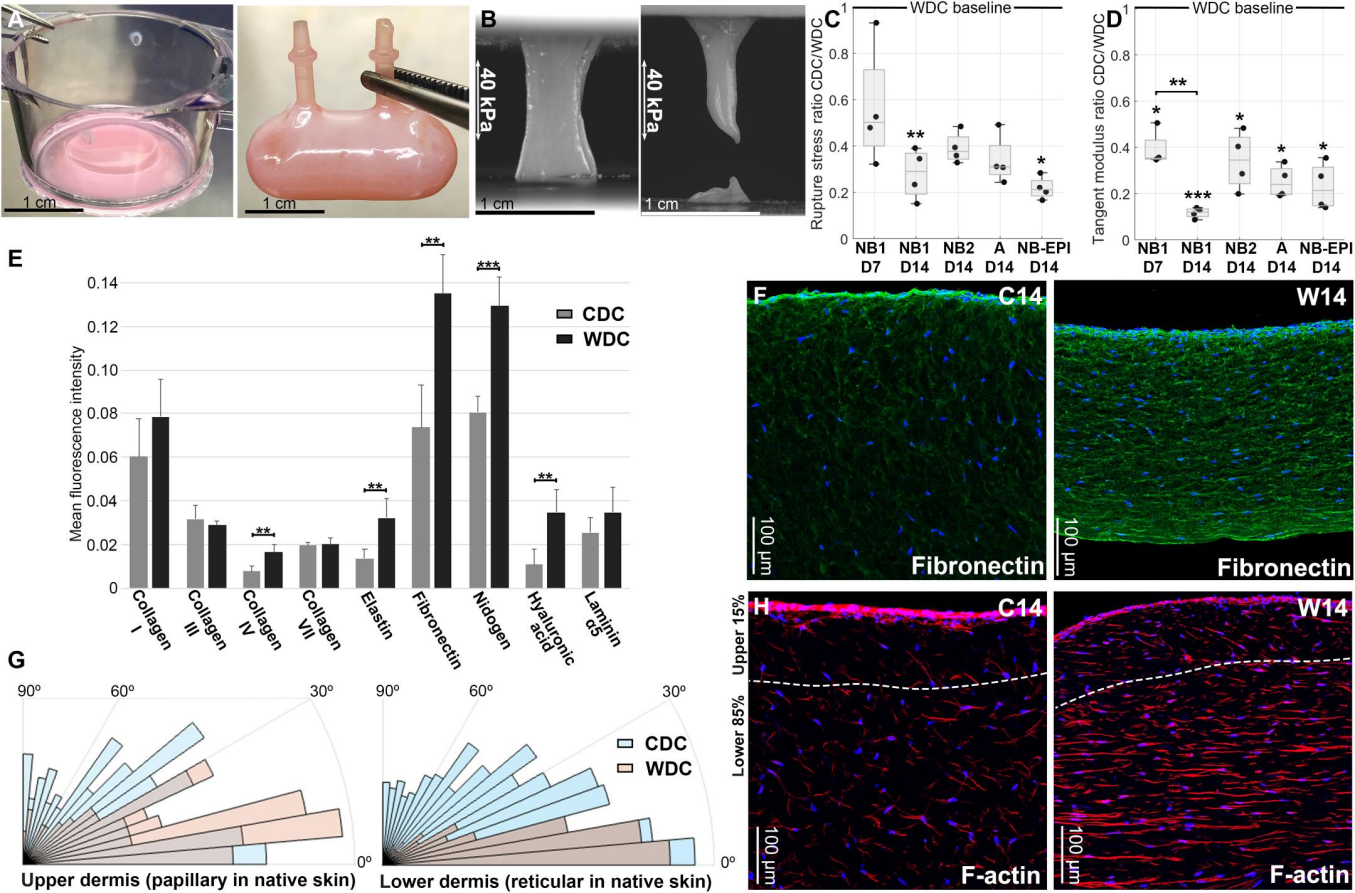
Analysis of the mechanical and morphological properties of the cylindrical WDCs in comparison to CDCs. (**A**) CDC on day 14 of culture (left); the construct contracts both in height and radius. Cylindrical WDC at day 14 (right); the WDC only decreases in thickness with no lateral contraction. (**B**) Photograms showing samples of WDC (left) and CDC (right) at a stress of 40 kPa during the uniaxial tension test. (**C**) Graph showing the ratio between CDC and WDC rupture stress within each condition [different time points and donors: newborn 1 (NB1), newborn 2 (NB2), adult (A), and NB2 with epidermis (NB-EPI)]. (**D**) Side-by-side comparison of the tangent modulus ratio between CDC and WDC within each condition. (**E**) Quantification of the mean fluorescence intensity (total intensity/pixel) for the principal constituents of ECM and BM (*P < 0.05, **P < 0.01, and ***P < 0.001). Collagen IV, elastin, fibronectin, nidogen, and hyaluronic acid are significantly up-regulated in the WDC. (**F**) IF staining demonstrating the up-regulation of fibronectin in the WDC at day 14. (**G**) Analysis of nuclei orientation in the upper 15% and lower 85% of the total thickness in CDC and WDC. (**H**) IF staining of F-actin fibers showing randomly oriented fibers throughout the CDCs and two different zones in the WDC: random organization in the upper quarter, similar to the papillary dermis, and thick horizontally aligned fibers in the lower 3/4th, resembling the reticular dermis. Data are based on four biological replicates for the CDCs and three biological replicates for the WDCs (two samples were cut from each WDC replicate). Scale bars, 1 cm (A and B) and 100 μm (F and H).

We tested the wearable edgeless dermal constructs (WDCs) side by side with conventional dermal constructs (CDCs) that we routinely produce and use in our studies (see Materials and Methods for the details for CDCs) (*1*–*3*, *25*, *26*). After 14 days of remodeling, the CDC was contracted, with 10 to 50% reduction in the original radius and roughly 80% in the thickness (Fig. 3A, left), while the WDC preserved its original length and width thanks to its enclosed geometry, only reducing its thickness from 4 to 0.5 mm (Fig. 3A, right). To assess the mechanical properties of the WDCs and CDCs, we cut out dog bone–shaped samples from the constructs and challenged the tissues with a uniaxial tension test (Fig. 3B). We observed marked differences in the stress-strain curves of WDCs and CDCs (fig. S6), with significantly higher rupture stresses achieved with WDCs. The CDCs showed no difference in rupture stress between day 7 and 14, while the WDCs almost doubled their rupture stress between these time points, displaying up to 400% higher rupture stress compared to the CDCs on day 14 in all the three donors tested (Fig. 3C), indicating an actively remodeled and mechanically evolving dermis. There was no significant difference between the rupture strain of CDCs and WDCs.

We additionally analyzed the stress-strain curve to calculate the tangent modulus and the low modulus as measures of the contribution of organized bundled (collagens I and III) and nonbundled ECM (e.g., elastin, which forms a disorganized network with random orientation), respectively, to the overall stiffness properties of the dermis (fig. S7, A and B). While the WDCs and CDCs did not exhibit a statistically significant difference in their low modulus properties (fig. S7C), the tangent modulus was significantly higher in WDCs with a 2.5-fold and 3- to 10-fold increase on days 7 and 14, respectively, compared to CDCs (Fig. 3D). In addition, we observed that the epidermis does not contribute to this enhancement in WDCs, since no significant difference could be detected between WDCs made with and without an epidermis (Fig. 3, C and D, and fig. S7). The individual J curves for each replicate tested at the uniaxial tension test can be found in figs. S8 and S9. These data suggested that the enhanced mechanical properties of the WDCs may derive from its enhanced fibrous ECM content and organization.

To investigate this possibility, we proceeded with IF staining of the dermal ECM. Although our starting ECM material was collagen I, after 14 days of remodeling, the FBs produced and deposited large amounts of other skin-relevant ECM proteins, such as collagen type III, fibronectin, and hyaluronic acid, closely matching the human dermis composition. Quantification of IF images showed that several key skin ECM molecules—such as collagen IV, elastin, hyaluronic acid, nidogen, and fibronectin—were significantly enhanced in WDCs compared to CDCs (Fig. 3, E and F, and figs. S10 and S11). When we checked the mRNA level, these ECM genes were not differentially regulated between WDCs and CDCs (fig. S12), suggesting that the improved mechanical properties of WDCs can be attributed to the enhanced ECM deposition and organization rather than the phenotypical changes in the dermal cells.

In the lower 85% portion of the dermal construct (which, in humans, corresponds to the reticular dermis) (*27*), the majority of the dermal FBs were parallel to the surface, with 39% of cells diverging <5° from the horizontal plane parallel to the surface, and were less branched compared to the upper 15% portion (which, in humans, corresponds to the papillary dermis) where only 11% of cells had <5° divergence from the horizontal plane, resembling the FB morphology in the reticular and papillary dermis of the human skin (Fig. 3, G and H). The distinction between the FB morphology was not present in CDCs where all FBs were randomly oriented throughout the dermis, with 10 and 11% of cells showing <5° of divergence from the horizontal plane, respectively. Moving from this observation, we proceeded to examine the expression of several FB markers, FB activation protein (FAP) and collagen XVIII (for papillary FBs) and CD90 (for reticular FBs) (*28*). FAP and collagen XVIII were expressed throughout the dermal thickness for both WDC and CDC, showing no preferential localization in the upper portion. The ratio of CD90^+^ cells located in the lower 85% portion to those in the upper 15% portion was higher in WDCs compared to CDCs on day 7 (roughly threefold higher in WDCs) (fig. S13). On day 14, CD90 expression became uniform in CDCs, whereas it was ~2 times higher in the lower portion of the WDCs than in the upper portion.

### Region-specific properties developed in the wearable edgeless skin

**Fig. 4.**
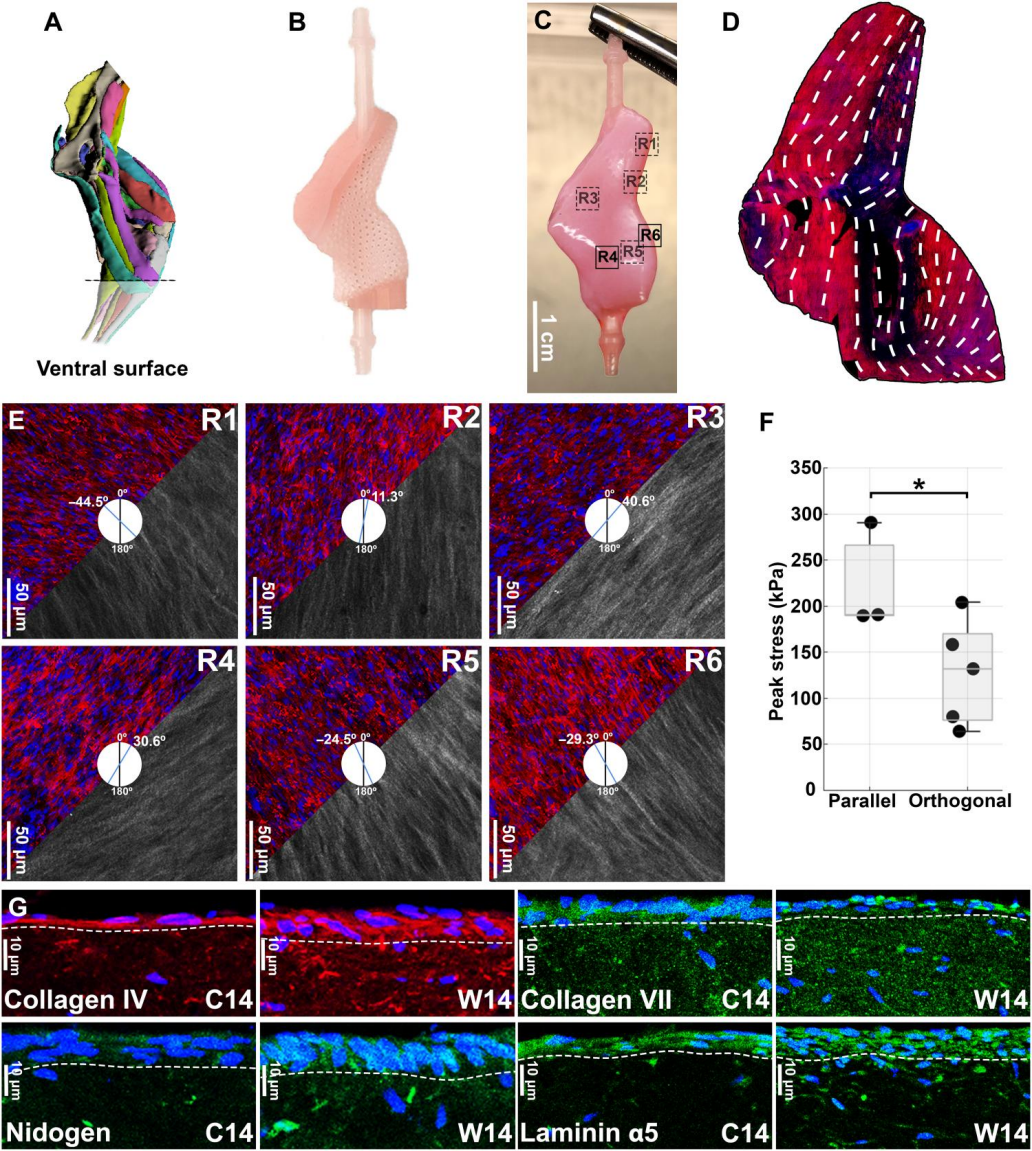
Analysis of the region-specific properties of the WHCs and comparison with CDCs. (**A**) CT scan of a murine left hindlimb after which the hindlimb scaffold was designed (**29**). (**B**) 3D-printed hindlimb scaffold. (**C**) WHC after 14 days of culture. (**D**) F-actin map of the ventral surface of a WHC showing the FB alignment, which indicates the directionality of tension forces. (**E**) 2GH microscopy and elastin IF of six different regions of a WHC: The collagen fibers and the elastic fibers run parallel. The blue vector at the center of the image indicates the median orientation of the nuclei. (**F**) Comparison of peak stress values of WHC samples stretched in the parallel and orthogonal directions in respect to FB alignment (*P < 0.05). (**G**) IF staining comparing side by side the expression of collagen IV, collagen VII, laminin α5, and nidogen in CDCs and WHCs at day 14 of culture. The data in (F) are based on six biological replicates, and two different regions were analyzed in parallel and orthogonal direction. The data in (D) and (E) are based on two biological replicates. The data in (G) are based on four biological replicates for the CDCs and six biological replicates for the WDCs. Scale bars, 1 cm (C), 50 μm (E), and 10 μm (G).

A notable feature of WDCs is that FBs form a multicellular layer with a high ECM density on the upper surface of the construct, where normally KCs are seeded to attach and produce the basement membrane (BM). On the basis of this observation, we examined the deposition and alignment of the ECM and cells on the upper surface to explore (i) the influence of this effect on the BM and (ii) the possibility of capturing region-specific structural differences found in human skin. To this end, we used the mouse hindlimb anatomy to serve as a more physiologically relevant geometry that could also be used in further transplantation studies in mice. We used a computerized tomography (CT) scan of a left mouse hindlimb (*29*) (Fig. 4A) as a blueprint to design the mouse hindlimb scaffold (Fig. 4B). We then used this scaffold to generate wearable mouse hindlimb constructs (WHCs) (Fig. 4C) following the aforementioned protocol, using the corresponding parts for the hindlimb design (Fig. 1A and figs. S1 and S2).

Moving from the observation of a different level of organization in the cylindrical WDCs compared to CDCs, we generated several dermal-only WHCs, performed whole-mount IF staining of F-actin fibers, and scanned the entire superficial layer of the WHCs for F-actin fluorescence to create a high-resolution cellular map (fig. S14). The F-actin organization on the ventral surface of dermal-only WHCs is shown in Fig. 4D, where the superimposed guidelines represent the general orientation of the fibers. We further characterized this self-organizational behavior through second harmonic generation (2GH) microscopy and elastin IF staining on six different regions of interest (Fig. 4E; regions shown in Fig. 4C) and quantified the cellular alignment in these regions. Notably, this analysis revealed region-specific alignment of dermal cells and their cytoskeleton on the dermal surface.

To interrogate whether the orientation of collagen fibers in different regions would lead to directional mechanical properties, we stretched the samples from two regions in the direction we expected to be either parallel or orthogonal to the orientation of the FBs and the ECM fibers and measured their stress over time (Fig. 4F). We found that the peak stress values were significantly higher (*P* < 0.025) when the WHCs were stretched in the direction parallel to the fibers, in alignment with the previously reported mechanical data obtained in respect to Langer lines for human skin (*30*). We also found that the WHCs were capable of reaching a higher strain with a lower level of stress when loaded orthogonally to the fibers’ orientation, in accordance to what was reported in literature (fig. S8) (*31*). Moreover, to understand to which extent the surface localization of dermal cells in wearable skin contributes to the deposition of BM proteins, we examined several critical BM proteins with IF imaging. All the main components of BM—such as collagen IV, collagen VII, laminin α5, and nidogen (Fig. 4G and fig. S11)—showed higher expression on the WHCs compared to CDCs, which may positively influence the development of a healthier epidermis and improve the outcome of the grafting.

Captivated by this up-regulation observed with IF, we analyzed this superficial cell layer in CDC and WDC with transmission electron microscopy (TEM) (fig. S15). On the dermal surface, CDCs show flattened FBs parallel to the surface and the presence of thick collagen fibers, likely collagen I or III (black arrowheads), as opposed to fibrillar BM proteins, e.g., collagen IV and laminin. This observation is in accordance with the IF staining that shows a significant collagen III up-regulation in the upper region of the CDCs (fig. S11). On the contrary, WDCs’ surface shows larger cells, containing a high number of secretion vesicles rich of thin fibrillar material and were surrounded by numerous exosomes. On the surface, WDCs present a thin layer of fibrillae of about 25 to 40 nm. To further investigate the functionality of this BM-like layer, we performed a KC attachment/retention assay (fig. S16). The WDC presented a higher level of cellular retention, i.e., fourfold higher KC density compared to CDCs, suggesting that the surface properties of the WDC may represent a more effective attachment substrate than those of the CDC counterpart.

### Vascularizing and transplanting wearable skin onto mice hindlimbs

**Fig. 5.**
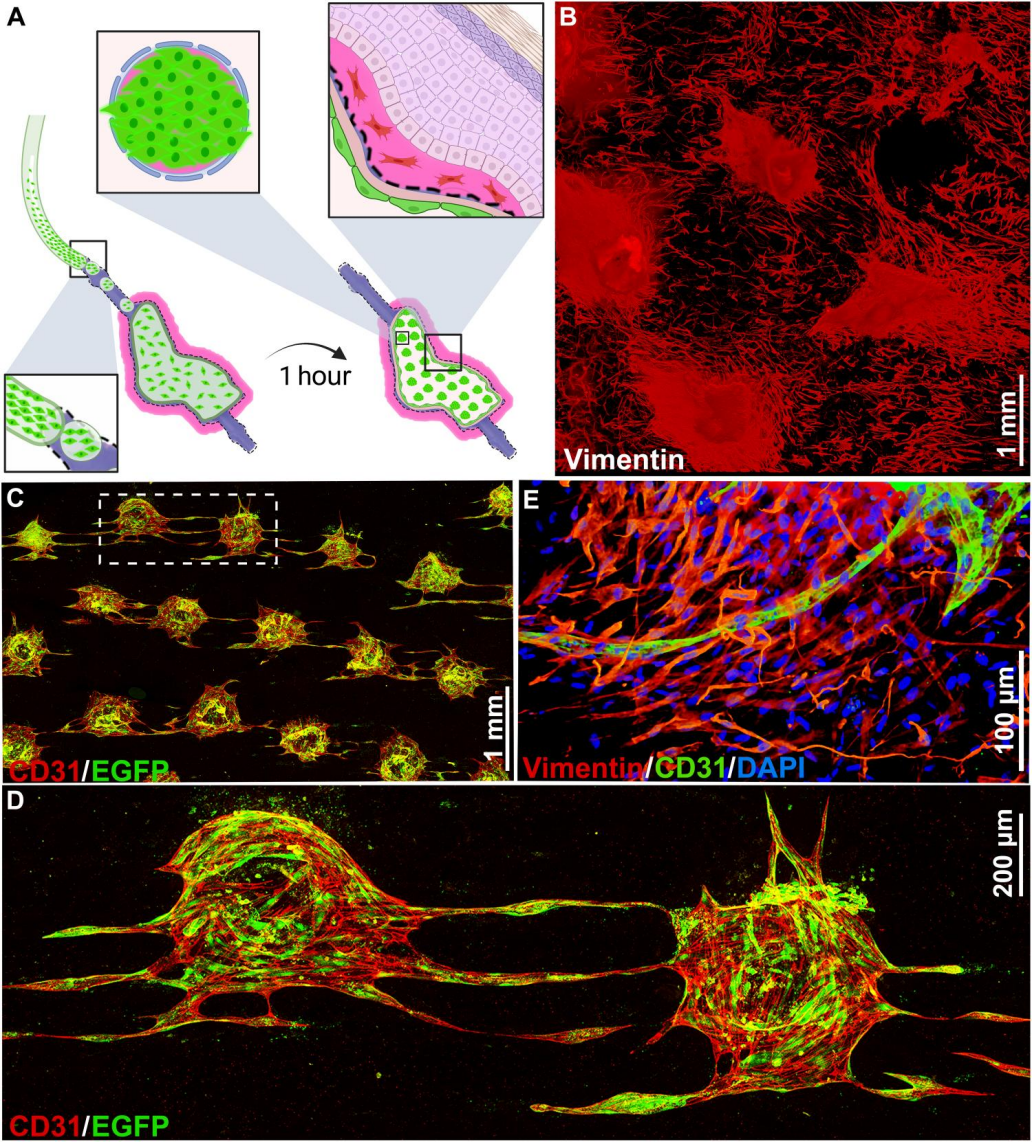
Vascularization of WHCs for further transplantation. (**A**) Illustration of EC seeding protocol in WHCs; the ECs are introduced inside the scaffold through injection and are allowed to attach for 1 hour on each surface before restarting the perfusion. (**B**) IF whole-mount staining showing FB and EC organization on the inner surface of the WHC; FBs and ECs are both stained with vimentin (red). (**C**) IF whole-mount staining showing the EC aggregates networking on the WHC inner surface. The EGFP-tagged HDBECs (green) were stained with CD31 (red), a specific endothelial surface marker. (**D**) Magnification of (C) showing the details of the aggregates sprouting toward each other; the spiral organization of the ECs in the aggregates fades into a very straight orientation in the sprouts. (**E**) IF staining showing a capillary surrounded by FBs; the capillary stained for CD31 (green) forms close interactions with the vimentin-positive FBs (red) and mimics their overall orientation. Scale bars, 1 mm (B and C), 200 μm (D), and 100 μm (E). Part of the illustration (A) was designed with Biorender.

We and others previously demonstrated the necessity of vascularizing engineered skin grafts for improved graft viability (*2*, *25*, *32*, *33*). In addition, our recent work highlighted the importance of using skin-specific endothelial cells (ECs) to vascularize engineered skin grafts (*25*). In light of these findings, we vascularized WESCs with human dermal blood ECs (HDBECs) before their engraftment onto mice hindlimbs. We seeded HDBECs through perfusion and let them attach on the inner walls of the dermis through the surface pores of the scaffold (Fig. 5A). To investigate the mechanism of vascular formation in WESCs, we cultured endothelialized WESCs in vitro for 7 days. The scaffold pores acted as microwells in which the ECs self-organized into round aggregates, interacting with the underlying mesh of FBs (the reticular region), which appear to irradiate from each aggregate and then organize as a network between the aggregates (Fig. 5B). The ECs sprouted from individual islands of aggregates to form interconnections (Fig. 5, C and D). The FBs were observed to wrap around the sprouting and elongated ECs (Fig. 5E and fig. S17). This mechanism of vessel-like formation in WESCs is similar to the cluster-based angiogenesis that was observed in vivo (*34*–*36*) and recently demonstrated in oxygen-controllable hydrogels (*37*). We also found that upon longer culture periods in vitro, the aggregates penetrate the dermal thickness and sprout toward the upper surface, reaching 480 μm along the *z* axis after 18 days, with individual HDBECs detectable up to 800 μm (fig. S18).

**Fig. 6.**
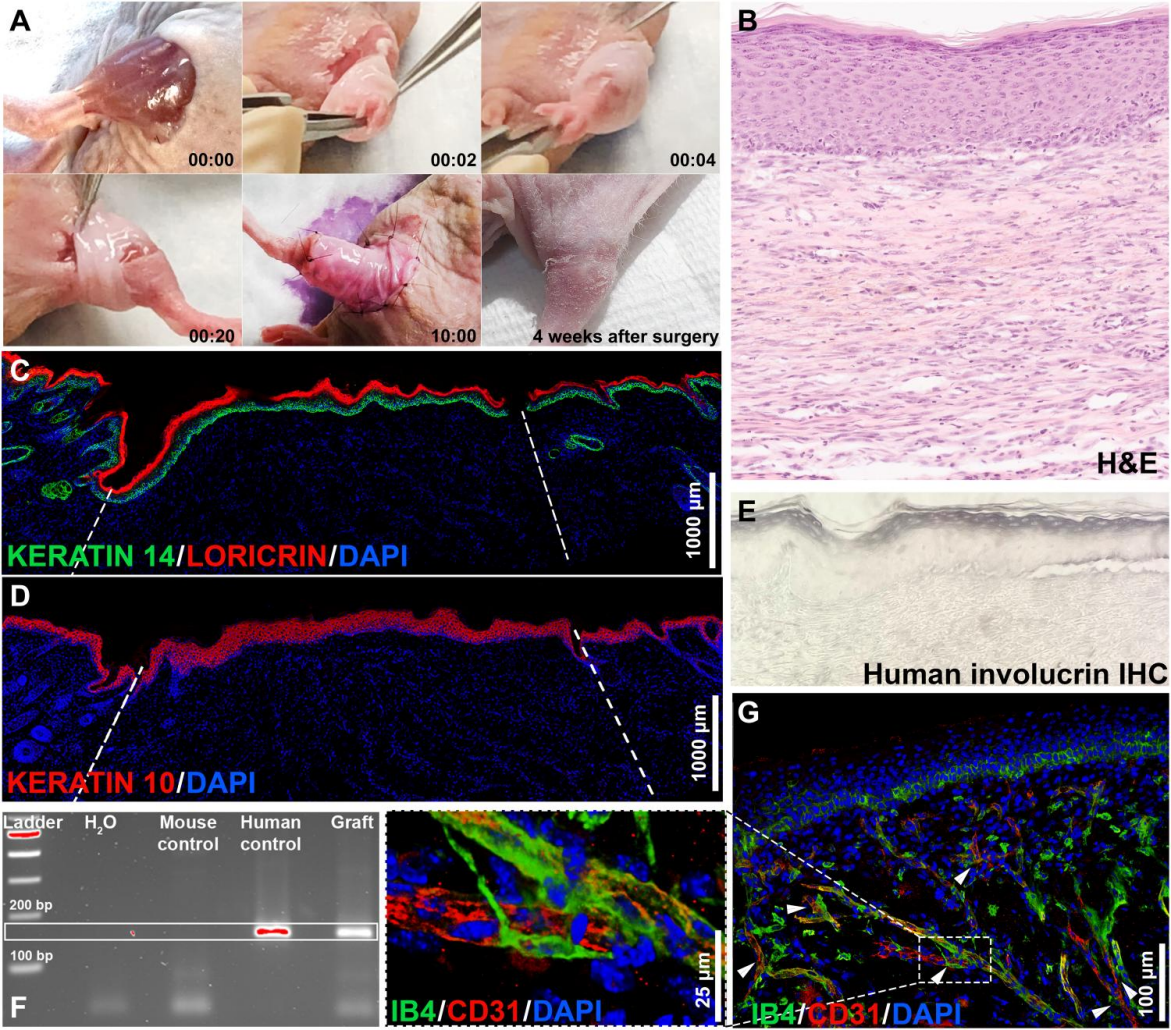
Transplantation of the vascularized WESCs onto mouse hindlimbs. (**A**) Time-lapse pictures of the grafting surgery and the integration after 4 weeks; the WESC is easily positioned as a sleeve and sutured to the adjacent mouse skin. Bottom right shows the integration of the graft after 4 weeks. (**B**) H&E staining showing the graft; the human epidermis is fully developed and cornified. (**C** and **D**) IF staining of the graft showing layer-specific epidermal markers of differentiation; Keratin 14, Keratin 10, and loricrin—markers of basal, suprabasal, and cornified layers, respectively—are expressed throughout the epidermis without major interruptions. (**E**) IHC staining for human-specific involucrin, demonstrating the presence of human skin tissue. (**F**) PCR for human-specific β-actin further confirming the survival of human cells in the graft. (**G**) IF staining showing the human (CD31^+^ IB4^−^) and mouse (CD31^+^ and IB4^+^) vasculature interaction and possible anastomosis sites (arrows). A total of 11 mice were used for grafting. Scale bars, 1000 μm (C and D), 100 μm (G), and 25 μm (G) (magnified).

To evaluate the transplantation capabilities of the wearable skin technology, the vascularized WESCs were removed from the hindlimb scaffold as a single piece and implanted onto the left hindlimb of nude mice. During the transplantation surgery, first, the mouse skin covering the hindlimb was excised from below the knee to the pelvis, and then WESC was placed seamlessly as a piece of clothing tailored specifically for the wound geometry (Fig. 6A and movie S2), keeping the duration of the graft placement under 40 s and the whole surgical procedure below 10 min for a large and difficult-to-graft wound area. We could not graft the same area with conventional skin constructs (CSCs) because of the excessive manipulation required to cut the circular CSC tissue into the shapes of interest, which would cause detachment of the epidermis. After 4 weeks from the surgery, the graft was completely integrated with the surrounding mouse skin (Fig. 6A, bottom right), and the mouse reacquired the full functionality of the lower limb (movie S3).

At this point, we harvested and analyzed the histology of the graft. The H&E staining in Fig. 6B shows the grafted WESCs, while the graft-host interface and normal mouse skin outside of the wound can be observed in fig. S19 (A and B, respectively). The IF shows an analog area with the graft at the center surrounded by the mouse skin (Fig. 6, C and D); the expression of Keratin 14, Keratin 10, and loricrin throughout the epidermis confirms full differentiation of the epidermis in the graft. To further differentiate the human epidermis from the murine epidermis, we performed immunohistochemistry (IHC) with a monoclonal antibody against human involucrin, which does not cross-react with the mouse (Fig. 6E), demonstrating the survival of the graft (the mouse skin control can be appreciated in fig. S19C). We also confirmed the presence of human cells through polymerase chain reaction (PCR) on DNA isolated from frozen tissue sections using primers for human-specific *β-actin* (Fig. 6F). Last, we explored the interaction between the human and the host vasculature through the double IF staining with a non–species-specific anti-CD31 and the rodent-specific griffonia simplicifolia isolectin B4 (Fig. 6G). The vessels were found to run in close proximity, with sporadic point of contact suggesting potential regions of anastomosis (arrows). Collectively, these data demonstrated the in vivo performance and translational potential of WESCs.

Next, we compared the viability and integration of prevascularized (*n* = 6) and nonvascularized (control, *n* = 5) WESCs. Although prevascularization is known to be effective for grafting CSCs on the back of small rodents and for deep human wounds (*1*, *2*, *25*, *32*, *33*, *38*, *39*), for WESCs grafted on the mouse hindlimbs, all grafts successfully integrated with the host regardless of their prevascularization status. Upon excision, the prevascularized graft showed higher blood content, which indirectly suggests higher oxygenation and nourishment of the graft, but analysis of cell proliferation (ki67) and cellular death [cleaved caspase 3 and terminal deoxynucleotidyl transferase–mediated deoxyuridine triphosphate nick end labeling (TUNEL), mostly absent] was comparable (fig. S20).

## DISCUSSION

Our study introduces wearable skin constructs as a compelling technology and provides a detailed description of a method to generate these tissues. Since 1981, when Bell *et al.* (*19*) presented the first full-thickness skin model as a planar tissue with open boundaries, primary research focus in the field has surrounded mimicking the cellular component of the bioengineered human skin, whereas the skin’s continuous and fully enclosed shape, e.g., the geometry component, has been a long-overlooked engineering variable. In this study, we demonstrated that simply remaining faithful to the enclosed geometry of the human skin improves dermal ECM deposition and organization, as well as mechanical properties, and notably presents body site–specific variations in cellular and extracellular organization in engineered 3D skin. Thanks to their enclosed and customizable shapes, these anatomically modeled 3D skin substitutes can be transplanted as a single wearable piece, minimizing the need for suturing, substantially reducing the length of surgeries, and improving the esthetic outcome by eliminating the scarring between multiple graft patches. In addition, the method allows to easily incorporate a vascular network, an important feature for the integration of the grafts onto the host, as we and others previously demonstrated (*2*, *25*, *32*, *33*). This technology does not require more cells than a conventional skin graft and therefore is clinically feasible to cover large areas. For instance, to graft the wearable skin on the hand of an adult male (mean surface area of 448 cm^2^), it would require nearly 45 million FBs (for 100,000 cells/cm^2^) and 18 million KCs (for 40,000 cells/cm^2^), which can be obtained from one 4-mm punch biopsy through in vitro expansion of KCs and FBs for four passages (we used up to passage #4 to make WESCs).

An interesting feature of WESCs is that FBs accumulate on the surface where they secrete densely packed ECM and elastin fibers with a specific orientation, which is greatly influenced by the shape of the scaffold. A similar migration and proliferation response of FBs on the surface of collagens was previously described as dependent on the presence of a full-length tensed fibronectin (*40*). The surface tension was also directly correlated with the proliferation of these cells on the growing edge of a wound healing model during tissue repair (*41*). The increased accumulation of FBs on the surface of WESCs is likely dependent on a similar mechanism, where the mechanical tension buildup enhances the FBs’ ability to secrete ECM, including fibronectin, generating a positive loop. Tensed fibronectin was reported to be prevalent in healthy tissue, while pathological transformations resulted in an increase in relaxed fibronectin (*42*); thus, it is possible that these qualities that WESCs have would have a protective influence on maintaining skin homeostasis. However, the mechanisms behind this cellular behavior in WESCs require further investigation.

We further explored whether the dense superficial area crowded by FBs on the top of the dermis could be considered a BM-like layer. The BM is a specialized structure, common to all epithelia, which is necessary to anchor the basal epithelial cells to the adjacent connective tissue. Both FBs and KCs contribute to the deposition of the BM, and most of its components are coexpressed by these two cell types, although some may be prevalently secreted by either one of them. A recent study highlighted that KCs are the major source of collagen XVII and some isoforms of laminin, while collagen IV and nidogen are mainly derived by FBs (*43*). We demonstrated that our dermal-only wearable constructs developed an abundant BM, containing many key components described for epidermal BMs in vivo. Some proteins such as collagen IV, laminin α5, and nidogen had a more tridimensional organization and slanted orientation, which resembles the way they distribute and orient in vivo. The current bioengineered skin constructs are known to have weak dermo-epidermal binding and BM expression, which is essential in maintaining the dermo-epidermal integrity as demonstrated by diseases in the epidermolysis bullosa (EB) spectrum, where the mutation of different proteins (mostly BM), such as collagen VII, leads to a spontaneous epidermal detachment following minimal mechanical insults. Here, we demonstrated that the surface of WDCs develops a BM-like structure with a thin nanoscale fibrillar network and improves KC attachment, compared to CDCs. In addition, WDC surface promotes an FB phenotype with large microvesicles and exosomes, although the specific content of these vesicular structures is yet to be investigated. We believe that the increased secretion of ECM and BM proteins in our WESCs can be greatly beneficial for treating patients with blistering skin diseases and can empower upcoming advanced cellular therapies (*44*–*47*). In addition, WESCs (e.g., skin gloves) would provide a personalized solution for difficult-to-transplant areas, such as the hands of EB patients with mitten-like deformities (*44*–*46*).

FBs are a heterogeneous group of cells of mesenchymal origin and morphology, which constitute connective tissues like the dermis (*48*, *49*). In the dermis, papillary FBs, which populate the superficial area of the dermis in close proximity to KCs, exhibit a spindle-shaped morphology, while reticular FBs, which reside deeper in the reticular part of the dermis and are main players in wound healing, appear squarer and stretched and show higher expression of smooth muscle actin (*50*). Recently, a growing body of work is revealing the molecular heterogeneity of different dermal FB subpopulations (*17*, *24*, *48*, *49*, *51*, *52*). In WESCs, we observed heterogeneous FB morphology across the dermis depth; in the upper dermis, the nuclei and F-actin show a tendency to orient randomly, while deeper in the tissue, the FBs look horizontally stretched and boast a morphology reminiscent of reticular FBs. Conversely, FBs in the CDCs were highly branched and randomly oriented throughout the construct’s depth. Although we examined the expression of several markers suggested for papillary and reticular FBs in WDCs, whether FB phenotypes are affected by the spatial biomechanical forces in WDCs remains inconclusive at this point, especially considering the fact that these markers for human FBs are still under debate in the literature (*53*).

The tension forces influencing the cellular and ECM alignment are also responsible for defining the pattern of ECM organization, e.g., Langer lines, in human skin (*54*). These lines are a consequence of the anatomical shape of the area and of the movements it is subjected to. Reconstructing the surface map of F-actin fiber orientation, which overlaps with collagen and elastic fibers, we demonstrated region-specific cellular and extracellular properties that depended on the anatomical site of the skin. Given the initial uniform cell density and dermal thickness, we believe that the shape of the scaffold is the major contributor in determining their directionality. Moving from this observation, we decided to test the effect of the fiber orientation on tissue mechanical resistance and found a significant difference when samples resected from the same areas were pulled in two orthogonal directions, resembling the anisotropic mechanical properties of human skin. This may translate into the clinic allowing for optimal mechanical adaptability of the graft based on the transplantation site at least at the time of grafting.

Notably, the WDCs exhibited a significantly increased mechanical resistance to rupture stress, compared to CDCs. In the analysis of the mechanical loading test, we calculated two important parameters: the tangent modulus, which reflects the engagement of organized structural fibers, such as collagen, and the low modulus, which represents the initial resistance to deformation generated by nonfibrous ECM before collagen fibers are straightened and engaged. We found that the tangent modulus was significantly higher in WDCs, which was consistent with the higher abundance and parallel organization of collagen fibers in the dermis. Although the density of other noncollagen ECM molecules, such as hyaluronic acid, was also increased in WDCs, this was not reflected in the low modulus values, which were similar for both types of constructs. This can be a result of the pronounced representation and dominant engagement of collagen bundles, especially in the reticular region of the dermis in WDCs, possibly masking the contribution of other ECM proteins. This observation is consistent with the mechanical properties of human skin where the tangent modulus is the dominant contributor of overall mechanical stiffness (*30*, *54*). It is important to note that these enhanced mechanical properties derive primarily by the enclosed fabrication method of the WDCs, since during the phase of dermal culture, the constructs are immersed in the medium and there is no active perfusion within the scaffold, resembling the same static culture conditions of CDCs. Nevertheless, dynamic culture during the later ALI phase may have effects on the homeostasis of the whole tissue, especially for the metabolically demanding KCs. The lack of contribution of the epidermis to the mechanical strength that we observed agrees with the mechanical properties of the human skin, in which tensile strength is dictated primarily by the dermis, given the thin epidermis layer compared to a much thicker dermis with a dense ECM network. These improved mechanical properties of WESCs allowed for seamless transplantation with easy handling during removal from the scaffold, graft placement, and suturing without any visible rupturing.

Our data suggest that WESC prevascularization is useful but not as essential as it is for conventional grafts typically performed on the back of mice. This can be attributed to several factors. First, the hindlimb is well vascularized, and the femoral artery (which, in our case, sprouted toward the grafts) runs in close proximity to the body surface. Second, WESCs made in our study are also considerably thinner than CSCs, which may facilitate the passive diffusion through the wound bed and the penetration of the vasculature up to the epidermis. In addition, rodents that have relatively fast cellular metabolism may promptly compensate for the lack of vasculature generating their own vascular network. Although, in humans, especially adults and elderly or not healthy individuals, cell metabolism is much slower (*55*), and thus, the presence of a vascular network can greatly enhance the success of skin (or any other) grafting, as extensively reported in literature (*1*, *2*, *25*, *32*, *33*, *38*). The prevascularization of WESCs would be especially useful in case of the so-called nongraftable wounds (*56*), where vascular insufficiency due to exposed bones, tendons, or thick scar tissue would prevent graft survival without a vascular bed. In this perspective, our data demonstrate the capability of WESCs to be effectively prevascularized before transplantation for these applications.

We believe that our advanced physiological skin constructs will have a marked impact on the future of skin bioengineering. This technology may not only transform the way skin grafts are generated but also influence other areas of research, such as drug development, cosmetics testing, and studies of fundamental skin biology. Our protocol allows for the generation of skin of highly complex geometries; here, we demonstrated the feasibility for the hands, but perhaps, another compelling application site would be the face. We envision that wearable skin can be integrated with other underlying tissues (cartilage, muscle, and bone) in the future and offer a personalized, autologous, and wearable alternative to vascularized composite allografts that are currently used to treat massive tissue defects, such as complete loss of facial features as a result of burn and trauma, revolutionizing composite tissue transplantation (e.g., face transplantation). Further research is needed to enhance the cellular complexity of the wearable constructs with skin appendages, pigmentation, and—in case of in vitro studies—a functional immune system. Despite these current limitations, we believe that the technology described here will greatly advance the field of skin bioengineering and has the potential to be a milestone in tissue engineering of soft tissues.

## MATERIALS AND METHODS

### Experimental design

We first developed a protocol to generate skin constructs in fully enclosed and anatomical shapes (WESCs), postulating that remaining faithful to the physiological geometry of the skin would influence their biological and mechanical properties. Then, we assessed the most important features of skin grafts such as (i) epidermal coverage, maturation, and permeability; (ii) the dermal composition, organization, and its mechanical properties; (iii) the potential for vascularization; and (iv) the feasibility for seamless transplantation onto an anatomically complex recipient site.

### Cell culture

Neonatal FBs and KCs from two newborns were isolated in our laboratory from foreskins donated to the Presbyterian Hospital (Columbia University Institutional Review Board protocol AAAB2666). Adult FBs (Promocell) were from the abdominal skin of a 75-year-old female. After isolation, cells were subcultured for three passages before being used in the experiments. FBs were cultured in Dulbecco’s modified Eagle’s medium (DMEM) with GlutaMAX and sodium pyruvate (Gibco, #10569010) supplemented with 10% fetal bovine serum (Gibco, #16000069) and antibiotic-antimycotic (Gibco, #15240062). KCs were cultured in collagen I peptide–coated dishes (Corning, #354450) with Epilife medium (Gibco, #MEPI500CA) supplemented with S7 (Gibco, #S0175). The normal adult HDBECs were purchased from Promocell (#C-12225, used for prevascularization of hindlimb grafts), while the green fluorescent protein (GFP)–tagged HDBECs were purchased from Angioproteomie (#cAP-0005GFP, used in vitro to assess the development of the blood vasculature in our model). The HDBECs were grown on dishes coated with Quick Coating Solution (Angioproteomie, #cAP-01) using endothelial growth medium MV (Promocell, #C-22120) and expanded for three passages before being used in the experiments. FBs and HDBECs were dissociated using trypsin-EDTA 0.05% (Gibco, #25300054), while Accutase (Gibco, #A1110501) was used for KCs.

### Fabrication of the scaffolds and the molds

The CAD models of body parts were obtained from online databases or literature (*29*) and converted into hollow shells with pores on the surface using Netfabb and nTopology softwares. The inlet and outlet ports of the scaffolds were designed and then added to the scaffold shelves using the Solidworks software. The scaffolds were 3D-printed using a Carbon DLS printer through Protolabs (Telford, UK) and CadBLU (New York, USA) with a biocompatible material, KeySplint Soft (keyprint). All scaffolds were designed to have one inlet and one outlet port for perfusion, a shell thickness of 0.7 mm, and 0.5-mm pores with an average pore-pore distance of 2 mm. The optimal pore diameter was dictated by practical exigences and the aim to achieve the largest possible interface area between the dermis and the medium present inside the scaffold. Pores with a diameter of >0.5 mm cause the culture medium to leak through the scaffold wall during perfusion, while the printing resolution of the 3D printer used would not allow to fabricate pores with a diameter of <0.1 mm. In addition, the rheological characteristics of the printing material after curing had to be taken into account to balance the pore density and wall thickness. Therefore, we adopted the highest pore density possible according to the aforementioned parameters. To generate the negative PDMS molds, the 3D model of each scaffold was enlarged with a 4-mm offset, divided in two parts on the horizontal plane roughly in the middle of the longitudinal axis, and 3D-printed in polylactic acid with a benchtop 3D printer (Sindoh 3DWOX). Subsequently, the positive print was enclosed in a 3D-printed chamber, which was filled with PDMS (Sylgard 184, Electron Microscopy Sciences, #24236-10) mixed with curing agent at a 10:1 ratio and cured at 60°C overnight. The molds were designed to have docking channels to accommodate the inlet and outlet port and stabilize the scaffolds in the proper position during WESC generation.

### Generation of the wearable edgeless skin

FBs were expanded up to passage #3 (P3), harvested with trypsin and resuspended in a neutralized and salt-balanced collagen solution with collagen type I (3 mg/ml; EMD Millipore, #08-115) at a density of 250 × 10^3^ cells/ml to form the dermis solution. The scaffold was plugged through the inlet/outlet ports in the docking channels of one mold, and then the two complementary molds were assembled together. The dermis solution was injected in the 4-mm space between the scaffold and the mold and incubated at 37°C for 1 hour for collagen polymerization, and then the scaffold bearing the dermis was removed from the mold and submerged in dermis medium. The volume of dermis solution used varies for each type of scaffold, with roughly 5, 6, and 12 ml for cylinder, hindlimb, and hand, respectively. The dermis was cultured for 14 days to allow contraction and remodeling, replacing the medium every other day before seeding the KCs. KCs at P3 were harvested when 90% confluent with Accutase and resuspended at 10 × 10^6^ cells/ml in EpilifeS7 medium. After removing the dermis medium, the construct was repositioned within the PDMS molds, and a KC solution of about 1 × 10^6^ cells/ml was injected into the mold and incubated on a rocking platform with a speed of 2 rpm. After 4 hours, the skin construct was removed from the mold and submerged in EPI medium (*18*). As an alternative method, in some constructs, the KC suspension was pipetted on the surface of the dermis, and the WESC was left to rest in the incubator for 30 min to allow KC attachment. The culture vessel was then filled with enough EPI medium to completely cover the WESC. The construct was further cultured for 5 to 7 days, changing the medium every other day. The cylinder and hindlimb constructs were seeded with 4 × 10^6^ KCs, while the hand construct was seeded with 8 × 10^6^ KCs. After epidermalization, the construct was seeded with ECs for the sprouting assay or directly switched to ALI culture with CORN medium (*18*) to allow terminal KC differentiation and cornification for all the other experiments. The grafts were seeded with ECs the day before the grafting in ALI culture conditions. For EC seeding, HDBECs 90% confluent at P3 were harvested with 0.5% trypsin and resuspended in coculture medium at a density of 4 × 10^6^ cells/ml. For the sprouting assay, the cylinder was positioned lying flat on one surface, and 500 μl of cell suspension was injected inside the scaffold through the perfusion port. After letting the cells settle for 1 hour, the cylinder was flipped, and an additional 500 μl was injected through the same perfusion port. After 1 hour, the construct was submerged in coculture medium and switched to ALI culture the following day. To seed the ECs in the to-be-grafted constructs, the procedure was similar; in this case, the constructs were first cultured in ALI for 2 days, then the perfusion was momentarily stopped, and the ECs were injected inside the construct through a valve in the perfusion system. The perfusion was restarted after 2 hours to allow EC attachment. To transition to ALI culture, the constructs were removed from the culture vessel and transferred to a 100-ml culture bottle with a custom-made lid that allowed the passage of the perfusion tubing and gas circulation. The constructs were suspended in the air hanging from the lid supported by the tubing. The medium was perfused with a multichannel peristaltic pump (Ismatec, #ISM936C) at a rate of 5 ml/min. The construct culture bottle was connected through the fluidic system with a reservoir bottle with analog features that contained the culture medium.

### Generation of CDCs

FBs were harvested at P3 with trypsin and resuspended in a neutralized and salt-balanced collagen solution with collagen type I (3 mg/ml; EMD Millipore, #08-115) at a density of 165 × 10^3^ cells/ml to form the dermis solution. To achieve uniform contraction, first, 1 ml of the neutralized collagen solution not containing cells was poured inside a transwell insert in a six-well plate (polyethylene terephthalate high-density 3-μm pores, Corning, #353092), then immediately after polymerization of the acellular layer, 3 ml of the dermis solution was layered on the top and incubated at 37°C for 15 min to allow for collagen polymerization, and then the culture plate and the transwell insert were filled in dermis medium to submerge the dermis. The dermis was cultured for 14 days to allow contraction and remodeling, replacing the medium every other day, after which the CDCs were used for downstream assays.

### Epidermal permeability assay with Lucifer yellow

We isolated each region of the hand skin construct with paraffin gel and sequentially applied 50 μl of Lucifer yellow dye solution (Invitrogen, #L453) at a concentration of 10 mg/ml with intervals of 1 hour between each region, collecting medium samples every 10 min. Dye permeation was quantified by measuring the fluorescent emission in the medium samples at 514 nm with the SpectraMax iD3 Multimode Microplate Reader (Molecular Devices). To avoid fluorescence from phenol red, we used phenol red–free DMEM and F12 as basal media to culture the samples tested in this assay.

### Numerical models

The glucose concentration and velocity profiles in WESCs were estimated by simultaneously solving for laminar flow and transport of diluted species modules in space using COMSOL Multiphysics software version 5.6. The 3D hand geometry was imported as a CAD model to COMSOL. The boundary conditions were taken as no-slip on the interior walls with an average glucose consumption rate of 4.2 × 10^–8^ mol/s per square meter based on a surface cell density of 5 × 10^5^ cells/cm^2^ and cellular glucose intake rate of 8.3 × 10^–9^ nmol/s per cell (*57*). The inlet velocity was changed as a variable in the range of the experimental perfusion rates used in the study, 0.1 to 5.0 ml/min, where the outlet condition was a static pressure of zero for a flow normal to the outlet opening. The initial glucose concentration was 25 mM, and diffusion coefficient was 9.59 × 10^–6^ cm^2^/s. The viscosity of the culture medium at 37°C was 0.78 × 10^–3^ Pa·s. The model was solved at the steady state with “Physics-controlled mesh” at a “coarser” element size. For the epidermal permeability simulations, the diffusion coefficient of Lucifer yellow was taken as 5 × 10^–10^ m^2^/s. The surface areas of permeation were 28, 45, and 30 mm^2^ for regions 1, 2, and 3, respectively. Epidermal permeability for each region was calculated by calculating the outlet dye concentration over time and fitting the experimental data by varying the dye permeability values.

### Mechanical property analysis

We used a customized steel die to cut dog bone–shaped samples from the tissue constructs. The gauge region of the specimen slice was 3 mm in length and 1.5 mm in width. This is to accommodate the small size of the constructs and, at the same time, create a uniform axial stress field at the sample center. Because the tissue construct bears residual stress and is compliant, the size of each testing sample varied slightly after cutting. For the CDCs, we cut one sample from each construct; for the WDCs, we cut two samples from each construct; in the cylindrical constructs, the samples were resected so that the main axis of the sample was parallel to the long axis of the cylinder, while in the hindlimb constructs, the samples were harvested from the dorsal surface with an angle of 45° between the long axes. Hence, we collected 4, 4, 6, 6, and 12 slices for the CDC day 7, CDC day 14, WDC cylinder day 7, WDC cylinder day 14, and WHC day 14, respectively. To study the anisotropic mechanical property, the samples for the WHC were cut in two directions, parallel and orthogonal to the limb’s proximal-distal orientation, resulting in two alignments of the loading direction relative to the fiber orientation, parallel and orthogonal. Immediately after cutting, we clamped the sample at its shoulder region of both ends using custom stainless-steel grips and mounted the setup onto a universal testing machine with a 10-N load cell (Instron Inc., Norwood, MA; accuracy of 0.01 N). We performed a two-part uniaxial tensile test following the protocol in a previous study (*58*). The first part is a 10-round cyclic test as preconditioning to provide the samples with a consistent loading history, where the sample was extended to a 10% peak strain in each cycle. The second part is a load-to-break test where the sample was extended until it ruptured (some slipped due to extreme thinning in the depth dimension, *n* = 1 for WDC cylinder day 7 and *n* = 2 for WDC cylinder day 14). In both parts, the tensile strain was prescribed as displacement, and the ramp rate was 1% of the gauge length per second. We used engineering strain in this study. Two orthogonal charge-coupled device cameras (Point Gray Grasshopper, GRAS-50S5M-C75 mm, f/4 lens) with light cast by LYKOS Daylight LED lights (Vitec Imaging Distribution Inc., Upper Saddle River, NJ) captured the sample’s dimension from the front and the side to record the real-time geometry. Force-time (N·s) data were recorded using a material tester software (Instron Inc., Blue Hill version 3.11.1209), and camera images were segmented in MATLAB. Calibration images were taken for each test set with a ruler of 1.58 mm gradations included in the field of view to convert all sample dimensions from the pixel field to millimeter. To avoid the variation in dimensions of each sample from affecting its strength performance, we calculated the true stress σ_true_ for our analysis. For each sample, we extracted its geometry using image segmentation, trimmed the top and bottom (25%) of the gauge region to eliminate the straining effect caused by the grips, and averaged the remaining section for its width and depth (width measured from the front and depth from the side). We multiplied the width and depth as the cross-sectional area (square millimeters) and used it to divide the force (newton) as the true stress σ_true_ (megapascal). We located the rupture point in time by finding the peak force and defined the corresponding stress and strain as the rupture stress and rupture strain. Peak stress is defined as the maximum stress observed during the test, and the corresponding strain is defined as the peak strain. For slipped samples, these values were defined as peak stress and peak strain instead. To assess the stiffness of the nonbundled ECM such as elastin and organized bundled (collagens I and III), we calculated the low modulus at a low strain (strain before the turning elbow in figs. S7 and S8) and the maximum tangent modulus *E*_max_ on the stress-strain curve by fitting a straight line tangent to the curve. All tangent moduli were calculated before rupture or slippage.

### Wearable hindlimb grafting onto mice

All experimental animal protocols were approved by the Institutional Animal Care and Use Committee at Columbia University Medical Center. After 3 days of ALI culture, the hindlimb constructs were grafted in the left limb of 8- to 12-week-old immunodeficient mice [males and females, weight of 20 to 30 g, athymic nude strain Crl:NU(NCr)-Foxn1^nu^, Charles River]. Eleven mice were used in this study. The mice were first anesthetized with isoflurane, and then the skin covering the left hindlimb of the mouse was removed using surgical scissors. At that point, the graft was removed from the incubator and brought to the surgery room. The excess tissue (upper and lower part of the hindlimb) was excised with a scalpel, and the graft was delicately removed from the scaffold with tweezers. The graft was then positioned in the recipient site, in a way that resembles wearing a pair of trousers—hence, we call them wearable skin constructs—and stitched to the adjacent skin. The total duration of the procedure was up to 15 min. A paraffin-embedded gauze was wrapped around the graft to avoid dehydration and secured to the site with additional bandages. The bandages were replaced after 4 days and removed after a week from the surgery, and a layer of paraffin gel was applied every day for an additional week. Mice were injected with carprofen (5 mg/ml per kilogram) before and after the procedure, followed by two additional administrations at 24 and 48 hours after the surgery. Four weeks following the grafting procedure, the mice were euthanized using CO_2_ followed by cervical dislocation, and then the grafts were harvested and processed for further analysis.

### Fluorescent staining and imaging of epidermis, ECM, and BM proteins

The presence of ECM and BM-related proteins was assessed with fluorescent confocal microscopy. The constructs and grafts were fixed in 4% paraformaldehyde in phosphate-buffered saline (PBS) overnight at 4°C, then transferred to a 30% sucrose solution for 24 hours at 4°C, and lastly embedded in optimal cutting temperature (O.C.T.) compound for cryosectioning (section thickness = 16 μm). For immunofluorescent staining, the slides were dried overnight at room temperature in the dark, then permeabilized with 0.1% Triton X, blocked with 8% bovine serum albumin and 5% donkey serum in PBS for 1 hour, and incubated overnight at 4°C with the following antibodies: keratin 14 (BioLegend, #906004), involucrin (Abcam, #ab27495), human-specific involucrin (Abcam, #ab68), loricrin (Abcam, #ab198994), filaggrin (Abcam, #ab221155), desmoglein 1 (Progen, #651111), desmoglein 3 (Invitrogen, #326300), collagen type I (Abcam, #ab6308), collagen type III (Abcam, #ab6310), collagen type IV (Abcam, #ab6586), collagen type VII (Abcam, #ab6312), collagen type XVIII (Santa Cruz Biotechnology, #sc32720), CD90 (BioLegend, #328110), FAP (R&D Systems, #AF3715-SP), smooth muscle actin (Abcam, #ab5694), fibronectin (Santa Cruz Biotechnology, #sc-73611), laminin α5 (EMD Millipore, #MABT39), nidogen (Abcam, #ab254325), elastin (Abcam, #ab21610), Ki67 (Abcam, #ab16667), and cleaved caspase 3 (Cell Signaling Technology, #9664). Different combinations of Alexa Fluor Plus secondary antibodies (Invitrogen) were used to detect the primary antibodies. Nuclei were stained with 4′,6-diamidino-2-phenylindole. Staining of the F-actin fibers was performed using phalloidin conjugated with Alexa Fluor 594 (Invitrogen, #A12381). Hyaluronic acid was stained using biotinylated versican G1 domain (Amsbio, #AMS.HKD-BC41) and Alexa Fluor 555–conjugated streptavidin (Invitrogen, S21381). To differentiate human and murine vasculature in tissue sections, we simultaneously stained the ECs with an anti-CD31 antibody (Abcam, #ab28364) directed both against human and mouse and with griffonia simplicifolia lectin I isolectin B4, DyLight 594 (Vector Laboratories, #DL-1207-.5), which stains the endothelium of several nonprimate animals including mice. 3D imaging of the vasculature was performed through whole-mount staining following the same protocol described for the tissue sections, with the exception of using 0.1% Triton X in all the staining and washing solutions. Anti-CD31 (Abcam, #ab28364) and anti-vimentin (Santa Cruz Biotechnology, #sc-6260) were used as primary antibodies. The tissue was cleared using Ce3D Tissue Clearing solution (BioLegend, #427704) for 20 min on a rocking platform before proceeding with the imaging. TUNEL assay for the detection of DNA fragmentation was performed on frozen sections of the grafts using Click-iT TUNEL Alexa Fluor 647 Imaging Assays for Microscopy & HCS (Thermo Fisher Scientific, #C10247) following the manufacturer’s recommendation. The images were acquired using a Leica Stellaris 5 (Leica, Germany). Three biological replicates were analyzed for each time point and sample.

### IHC staining

IHC staining was performed on frozen sections of the grafts, following antigen retrieval for 10 minutes in citrate buffer (pH 6). The primary antibody anti-human involucrin (Abcam, #Ab68) required the use of a mouse-on-mouse blocking kit (Vector Laboratories, #BMK-2202), which was used according to the manufacturer’s recommendation, including inhibition of endogenous peroxidases with 1% H_2_O_2_ incubation for 10 minutes and blocking of the endogenous biotin (Avidin/Biotin Blocking Kit, Vector Laboratories, #SP-2001). Staining was developed with the VECTASTAIN Elite ABC-HRP (horseradish peroxidase) Kit (Vector Laboratories, #PK-6200) and the DAB Substrate Kit HRP with nickel chloride (Vector Laboratories, #SK-4100). The images were acquired with a Leica SCN400.

### KC fluorescent labeling and attachment/retention test

Neonatal KCs at P3 were fluorescently tagged with CellTracker Deep Red Dye (Thermo Fisher Scientific, #C34565) following the manufacturer’s recommendations, with an incubation time of 40 min and a dye concentration of 1 μM, and resuspended in medium at a density of 5 × 10^6^/ml. After removing the culture medium, 10 μl of KCs suspension was applied on top of WDCs and CDCs on day 14 of culture. The constructs were incubated for 5 min, and then they were gently dipped in prewarmed DMEM/F12 basal medium (500 ml) for three times to remove nonattached cells. The constructs were positioned on a coverslip and immediately analyzed with a confocal microscope. The resulting images were analyzed with the software CellProfiler to quantify the cell number per square millimeter.

### Transmission electron microscopy

For TEM, the CDC and WDC samples were fixed at day 14 of culture in a buffer containing 4% paraformaldehyde, 2.5% glutaraldehyde, sodium cacodylate (0.1 M), and 1% tannic acid for 24 hours. The samples were subsequently embedded into resin and sectioned at a thickness of 70 nm. The sections were stained with osmium tetroxide and imaged with a JEOL1400 Flash TEM (Jeol) with a Gatan 4 k × 4 k Rio complementary metal-oxide semiconductor camera.

### Quantification of ECM and BM RNA expression

The expression of RNA transcripts for ECM and BM-related proteins was investigated with real-time quantitative PCR (qPCR). RNA was extracted by digesting the tissue with IBI isolate (IBI Scientific, #IB47602) followed by chloroform-based removal of the phenol. The RNA was then isolated with the RNeasy Mini Kit (Qiagen, #74104) and retrotranscribed to complementary DNA with the SuperScript IV VILO Kit (Invitrogen, #11766050). qPCR was performed on a CFX96 thermocycler (Bio-Rad) using TaqMan Fast Advanced Master Mix (Invitrogen, #4444556) and the following TaqMan probes: *COL1A2*-Hs01028956_m1, *COL3A1*-Hs00943809_m1, *COL4A1*-Hs00266237_m1, *COLVIIA1*-Hs00164310_m1, *LAMA3*-Hs00165042_m1, *LAMA5*-Hs00966585_m1, *FN1*-Hs01549976_m1, and *GAPDH*-Hs99999905_m1. The expression of target genes was normalized using glyceraldehyde-3-phosphate dehydrogenase (*GAPDH*) as a housekeeper gene, and the results were compared using the delta-delta CT formula. Three biological replicates were analyzed for each time point and sample, and qPCR was performed with three technical replicates per sample.

### Polymerase chain reaction

DNA was isolated from frozen sections of the grafts through crude extraction, digesting the sample in 50 mM NaOH at 95°C for 10 min, which was then neutralized with 1 M tris-HCl (pH 8.0). The control DNA was isolated from cell suspension of primary human FBs and mouse embryonic stem cells with the same method. The primers, targeting the gene b-actin, were designed with the software Primer-BLAST from the National Institutes of Health to amplify human-specific [CGCGGCGATATCATCATCCA (forward) and CGGCTTCCTTTGTCCCCAAT (reverse)] and mouse-specific sequences [TCCGCCTAGAAGCACTTGCG (forward) and CAGAGAGCTCACCATTCACCAT (reverse)]. The reaction used 45 μl of MasterMix (Terra PCR Direct Polymerase Mix, Takara Bio #639270), 5 μl of DNA, and primers at a concentration of 300 nM and was performed with a Bio-Rad CFX96 over 35 cycles.

### Statistical methods

The in vitro studies were conducted using a minimum of three biological replicates, with newborn 1 (NB1) CDCs *n* = 4, NB1 cylindrical WDCs *n* = 3 (with two technical replicates for the stretch test), NB1 hindlimb WDCs *n* = 6 (with two technical replicates for the stretch test), NB2 CDC *n* = 4, NB2 CSC *n* = 4, NB2 WDC *n* = 4, NB2 WESC *n* = 4, A CDC *n* = 4, and A WDC *n* = 4. Using the software G*Power, we calculated that a total of 10 mice were required to conduct the in vivo studies (point biserial correlation, effect size of 0.8, 𝜶 of 0.05, and power of 0.9566). A total of 11 mice were used (5 nonvascularized controls and 6 vascularized constructs). The mice were divided between male and females (six and five, respectively) and randomly selected for grafting with nonprevascularized or prevascularized WESC using the function “= RAND()” in Microsoft Excel. Inclusion and exclusion criteria, beside the aforementioned animal characteristics (strain and age), were limited to apparent good health (inclusion) and signs of distress (exclusion). The study was blinded to personnel that performed surgeries, animal care, and analysis (randomizing sample order for the analysis). All assays were repeated at least in triplicate, and the data are presented as the mean − SD. Shapiro-Wilk normality test and one-way analysis of variance (ANOVA) test (*P* < 0.05) were performed in Prism (Prism Software, Irvine, CA) to establish statistical significance between groups for the tangent modulus, low modulus, and stress. The qPCR data were analyzed through the software Prism 8 by using multiple unpaired *t* tests, not assuming a consistent SD for all rows. For all statistical analyses, *P* < 0.05 was considered significantly different, where *P < 0.05, **P < 0.01, ***P < 0.001, and ****P < 0.0001.

### Adherence to community standards

The experimental setup, data reporting, and manuscript writing were performed in accordance with Animal Research: Reporting of In Vivo Experiments (ARRIVE) guidelines.

## Notes

View/request a protocol for this paper from *Bio-protocol*.

## References

[1] H. E. Abaci, A. Coffman, Y. Doucet, J. Chen, J. Jackow, E. Wang, Z. Y. Guo, J. U. Shin, C. A. Jahoda, A. M. Christiano, Tissue engineering of human hair follicles using a biomimetic developmental approach. Nat. Commun. 9, 5301 (2018). 30546011 10.1038/s41467-018-07579-y PMC6294003

[2] H. E. Abaci, Z. Guo, A. Coffman, B. Gillette, W. H. Lee, S. K. Sia, A. M. Christiano, Human skin constructs with spatially controlled vasculature using primary and iPSC-derived endothelial cells. Adv. Healthc. Mater. 5, 1800–1807 (2016). 27333469 10.1002/adhm.201500936 PMC5031081

[3] J. U. Shin, H. E. Abaci, L. Herron, Z. Guo, B. Sallee, A. Pappalardo, J. Jackow, E. H. C. Wang, Y. Doucet, A. M. Christiano, Recapitulating T cell infiltration in 3D psoriatic skin models for patient-specific drug testing. Sci. Rep. 10, 4123 (2020). 32139717 10.1038/s41598-020-60275-0 PMC7057979

[4] K. Gledhill, Z. Guo, N. Umegaki-Arao, C. A. Higgins, M. Itoh, A. M. Christiano, Melanin transfer in human 3D skin equivalents generated exclusively from induced pluripotent stem cells. PLOS ONE 10, e0136713 (2015). 26308443 10.1371/journal.pone.0136713 PMC4550351

[5] D. M. Supp, J. M. Hahn, C. M. Lloyd, K. A. Combs, V. B. Swope, Z. Abdel-Malek, S. T. Boyce, Light or dark pigmentation of engineered skin substitutes containing melanocytes protects against ultraviolet light-induced DNA damage in vivo. J. Burn Care Res. 41, 751–760 (2020). 32052834 10.1093/jbcr/iraa029

[6] I. J. Kosten, S. W. Spiekstra, T. D. de Gruijl, S. Gibbs, MUTZ-3 derived Langerhans cells in human skin equivalents show differential migration and phenotypic plasticity after allergen or irritant exposure. Toxicol. Appl. Pharmacol. 287, 35–42 (2015). 26028481 10.1016/j.taap.2015.05.017

[7] M. Blais, L. Mottier, M. A. Germain, S. Bellenfant, S. Cadau, F. Berthod, Sensory neurons accelerate skin reepithelialization via substance P in an innervated tissue-engineered wound healing model. Tissue Eng. Part A 20, 2180–2188 (2014). 24716723 10.1089/ten.tea.2013.0535 PMC4137331

[8] C. A. Lopez Valle, L. Germain, M. Rouabhia, W. Xu, R. Guignard, F. Goulet, F. A. Auger, Grafting on nude mice of living skin equivalents produced using human COLLAGENS1,2. Transplantation 62, 317–323 (1996). 8779676 10.1097/00007890-199608150-00003

[9] A. Smith, T. Watkins, G. Theocharidis, I. Lang, M. Leschinsky, A. Maione, O. Kashpur, T. Raimondo, S. Rahmani, J. Baskin, D. Mooney, A. Veves, J. Garlick, A novel three-dimensional skin disease model to assess macrophage function in diabetes. Tissue Eng. Part C Methods 27, 49–58 (2021). 33280487 10.1089/ten.tec.2020.0263 PMC8349718

[10] T. Baltazar, J. Merola, C. Catarino, C. B. Xie, N. C. Kirkiles-Smith, V. Lee, S. Hotta, G. Dai, X. Xu, F. C. Ferreira, W. M. Saltzman, J. S. Pober, P. Karande, Three dimensional bioprinting of a vascularized and perfusable skin graft using human keratinocytes, fibroblasts, pericytes, and endothelial cells. Tissue Eng. Part A 26, 227–238 (2020). 31672103 10.1089/ten.tea.2019.0201 PMC7476394

[11] B. S. Kim, G. Gao, J. Y. Kim, D. W. Cho, 3D cell printing of perfusable vascularized human skin equivalent composed of epidermis, dermis, and hypodermis for better structural recapitulation of native skin. Adv. Healthc. Mater. 8, e1801019 (2019). 30358939 10.1002/adhm.201801019

[12] S. Kimura, A. Tsuchiya, M. Ogawa, M. Ono, N. Suda, K. Sekimoto, M. Takeo, T. Tsuji, Tissue-scale tensional homeostasis in skin regulates structure and physiological function. Commun. Biol. 3, 637 (2020). 33127987 10.1038/s42003-020-01365-7 PMC7603398

[13] R. Gauvin, T. Ahsan, D. Larouche, P. Levesque, J. Dube, F. A. Auger, R. M. Nerem, L. Germain, A novel single-step self-assembly approach for the fabrication of tissue-engineered vascular constructs. Tissue Eng. Part A 16, 1737–1747 (2010). 20038201 10.1089/ten.TEA.2009.0313

[14] Q. Muller, M. J. Beaudet, T. De Serres-Berard, S. Bellenfant, V. Flacher, F. Berthod, Development of an innervated tissue-engineered skin with human sensory neurons and Schwann cells differentiated from iPS cells. Acta Biomater. 82, 93–101 (2018). 30316025 10.1016/j.actbio.2018.10.011

[15] J. Bourland, J. Fradette, F. A. Auger, Tissue-engineered 3D melanoma model with blood and lymphatic capillaries for drug development. Sci. Rep. 8, 13191 (2018). 30181613 10.1038/s41598-018-31502-6 PMC6123405

[16] M. Huang, A. Smith, M. Watson, R. Bhandari, L. M. Baugh, I. Ivanovska, T. Watkins, I. Lang, M. Trojanowska, L. D. Black III, P. A. Pioli, J. Garlick, M. L. Whitfield, Self-assembled human skin equivalents model macrophage activation of cutaneous fibrogenesis in systemic sclerosis. Arthritis Rheumatol. 74, 1245–1256 (2022). 35212485 10.1002/art.42097 PMC9340654

[17] S. Mascharak, H. E. desJardins-Park, M. T. Longaker, Fibroblast heterogeneity in wound healing: Hurdles to clinical translation. Trends Mol. Med. 26, 1101–1106 (2020). 32800679 10.1016/j.molmed.2020.07.008

[18] P. Gangatirkar, S. Paquet-Fifield, A. Li, R. Rossi, P. Kaur, Establishment of 3D organotypic cultures using human neonatal epidermal cells. Nat. Protoc. 2, 178–186 (2007). 17401352 10.1038/nprot.2006.448

[19] E. Bell, H. P. Ehrlich, D. J. Buttle, T. Nakatsuji, Living tissue formed in vitro and accepted as skin-equivalent tissue of full thickness. Science 211, 1052–1054 (1981). 7008197 10.1126/science.7008197

[20] S. T. Boyce, R. J. Kagan, D. G. Greenhalgh, P. Warner, K. P. Yakuboff, T. Palmieri, G. D. Warden, Cultured skin substitutes reduce requirements for harvesting of skin autograft for closure of excised, full-thickness burns. J. Trauma 60, 821–829 (2006). 16612303 10.1097/01.ta.0000196802.91829.cc

[21] Y. Jiang, R. Guo, S. Zhou, B. Wang, W. Sun, Functional and aesthetic reconstruction of digital flexion contractures with full-thickness plantar skin grafts in children. Dermatol. Ther. 33, e14466 (2020). 33112495 10.1111/dth.14466

[22] L. C. Biggs, C. S. Kim, Y. A. Miroshnikova, S. A. Wickstrom, Mechanical forces in the skin: Roles in tissue architecture, stability, and function. J. Invest. Dermatol. 140, 284–290 (2020). 31326398 10.1016/j.jid.2019.06.137

[23] H. Joodaki, M. B. Panzer, Skin mechanical properties and modeling: A review. Proc. Inst. Mech. Eng. H 232, 323–343 (2018). 29506427 10.1177/0954411918759801

[24] R. R. Driskell, F. M. Watt, Understanding fibroblast heterogeneity in the skin. Trends Cell Biol. 25, 92–99 (2015). 25455110 10.1016/j.tcb.2014.10.001

[25] A. Pappalardo, L. Herron, D. E. Alvarez Cespedes, H. E. Abaci, Quantitative evaluation of human umbilical vein and induced pluripotent stem cell-derived endothelial cells as an alternative cell source to skin-specific endothelial cells in engineered skin grafts. Adv. Wound Care (New Rochelle) 10, 490–502 (2020). 32870778 10.1089/wound.2020.1163 PMC8260893

[26] H. E. Abaci, K. Gledhill, Z. Guo, A. M. Christiano, M. L. Shuler, Pumpless microfluidic platform for drug testing on human skin equivalents. Lab Chip 15, 882–888 (2015). 25490891 10.1039/c4lc00999a PMC4305008

[27] V. Marcos-Garces, P. Molina Aguilar, C. Bea Serrano, V. Garcia Bustos, J. Benavent Segui, A. Ferrandez Izquierdo, A. Ruiz-Sauri, Age-related dermal collagen changes during development, maturation and ageing - a morphometric and comparative study. J. Anat. 225, 98–108 (2014). 24754576 10.1111/joa.12186 PMC4089350

[28] A. Korosec, S. Frech, B. Gesslbauer, M. Vierhapper, C. Radtke, P. Petzelbauer, B. M. Lichtenberger, Lineage identity and location within the dermis determine the function of papillary and reticular fibroblasts in human skin. J. Invest. Dermatol. 139, 342–351 (2019). 30179601 10.1016/j.jid.2018.07.033

[29] J. P. Charles, O. Cappellari, A. J. Spence, J. R. Hutchinson, D. J. Wells, Musculoskeletal geometry, muscle architecture and functional specialisations of the mouse hindlimb. PLOS ONE 11, e0147669 (2016). 27115354 10.1371/journal.pone.0147669 PMC4846001

[30] X. Liang, S. A. Boppart, Biomechanical properties of in vivo human skin from dynamic optical coherence elastography. I.E.E.E. Trans. Biomed. Eng. 57, 953–959 (2010). 10.1109/TBME.2009.2033464 PMC3699319 19822464

[31] A. B. Tepole, A. K. Gosain, E. Kuhl, Stretching skin: The physiological limit and beyond. Int. J. Non Linear Mech. 47, 938–949 (2012). 23459410 10.1016/j.ijnonlinmec.2011.07.006 PMC3583021

[32] F. S. Frueh, T. Spater, C. Korbel, C. Scheuer, A. C. Simson, N. Lindenblatt, P. Giovanoli, M. D. Menger, M. W. Laschke, Prevascularization of dermal substitutes with adipose tissue-derived microvascular fragments enhances early skin grafting. Sci. Rep. 8, 10977 (2018). 30030486 10.1038/s41598-018-29252-6 PMC6054621

[33] L. Chen, Q. Xing, Q. Zhai, M. Tahtinen, F. Zhou, L. Chen, Y. Xu, S. Qi, F. Zhao, Pre-vascularization enhances therapeutic effects of human mesenchymal stem cell sheets in full thickness skin wound repair. Theranostics 7, 117–131 (2017). 28042321 10.7150/thno.17031 PMC5196890

[34] K. Proulx, A. Lu, S. Sumanas, Cranial vasculature in zebrafish forms by angioblast cluster-derived angiogenesis. Dev. Biol. 348, 34–46 (2010). 20832394 10.1016/j.ydbio.2010.08.036

[35] O. M. Tepper, J. M. Capla, R. D. Galiano, D. J. Ceradini, M. J. Callaghan, M. E. Kleinman, G. C. Gurtner, Adult vasculogenesis occurs through in situ recruitment, proliferation, and tubulization of circulating bone marrow-derived cells. Blood 105, 1068–1077 (2005). 15388583 10.1182/blood-2004-03-1051

[36] P. Vajkoczy, S. Blum, M. Lamparter, R. Mailhammer, R. Erber, B. Engelhardt, D. Vestweber, A. K. Hatzopoulos, Multistep nature of microvascular recruitment of ex vivo-expanded embryonic endothelial progenitor cells during tumor angiogenesis. J. Exp. Med. 197, 1755–1765 (2003). 12810693 10.1084/jem.20021659 PMC2193947

[37] M. R. Blatchley, F. Hall, S. Wang, H. C. Pruitt, S. Gerecht, Hypoxia and matrix viscoelasticity sequentially regulate endothelial progenitor cluster-based vasculogenesis. Sci. Adv. 5, eaau7518 (2019). 30906859 10.1126/sciadv.aau7518 PMC6426463

[38] H. Miyazaki, Y. Tsunoi, T. Akagi, S. Sato, M. Akashi, D. Saitoh, A novel strategy to engineer pre-vascularized 3-dimensional skin substitutes to achieve efficient, functional engraftment. Sci. Rep. 9, 7797 (2019). 31127144 10.1038/s41598-019-44113-6 PMC6534548

[39] M. A. Nilforoushzadeh, M. M. Sisakht, M. A. Amirkhani, A. M. Seifalian, H. R. Banafshe, J. Verdi, M. Nouradini, Engineered skin graft with stromal vascular fraction cells encapsulated in fibrin-collagen hydrogel: A clinical study for diabetic wound healing. J. Tissue Eng. Regen. Med. 14, 424–440 (2020). 31826321 10.1002/term.3003

[40] J. Foolen, J. Y. Shiu, M. Mitsi, Y. Zhang, C. S. Chen, V. Vogel, Full-length fibronectin drives fibroblast accumulation at the surface of collagen microtissues during cell-induced tissue morphogenesis. PLOS ONE 11, e0160369 (2016). 27564551 10.1371/journal.pone.0160369 PMC5001707

[41] P. Kollmannsberger, C. M. Bidan, J. W. C. Dunlop, P. Fratzl, V. Vogel, Tensile forces drive a reversible fibroblast-to-myofibroblast transition during tissue growth in engineered clefts. Sci. Adv. 4, eaao4881 (2018). 29349300 10.1126/sciadv.aao4881 PMC5771696

[42] C. M. Fonta, S. Arnoldini, D. Jaramillo, A. Moscaroli, A. Oxenius, M. Behe, V. Vogel, Fibronectin fibers are highly tensed in healthy organs in contrast to tumors and virus-infected lymph nodes. Matrix Biol. Plus 8, 100046 (2020). 33543039 10.1016/j.mbplus.2020.100046 PMC7852196

[43] K. Tsutsui, H. Machida, A. Nakagawa, K. Ahn, R. Morita, K. Sekiguchi, J. H. Miner, H. Fujiwara, Mapping the molecular and structural specialization of the skin basement membrane for inter-tissue interactions. Nat. Commun. 12, 2577 (2021). 33972551 10.1038/s41467-021-22881-y PMC8110968

[44] T. Hirsch, T. Rothoeft, N. Teig, J. W. Bauer, G. Pellegrini, L. De Rosa, D. Scaglione, J. Reichelt, A. Klausegger, D. Kneisz, O. Romano, A. Secone Seconetti, R. Contin, E. Enzo, I. Jurman, S. Carulli, F. Jacobsen, T. Luecke, M. Lehnhardt, M. Fischer, M. Kueckelhaus, D. Quaglino, M. Morgante, S. Bicciato, S. Bondanza, M. De Luca, Regeneration of the entire human epidermis using transgenic stem cells. Nature 551, 327–332 (2017). 29144448 10.1038/nature24487 PMC6283270

[45] N. Umegaki-Arao, A. M. Pasmooij, M. Itoh, J. E. Cerise, Z. Guo, B. Levy, A. Gostynski, L. R. Rothman, M. F. Jonkman, A. M. Christiano, Induced pluripotent stem cells from human revertant keratinocytes for the treatment of epidermolysis bullosa. Sci. Transl. Med. 6, 264ra164 (2014). 10.1126/scitranslmed.3009342 25429057

[46] V. Sebastiano, H. H. Zhen, B. Haddad, E. Bashkirova, S. P. Melo, P. Wang, T. L. Leung, Z. Siprashvili, A. Tichy, J. Li, M. Ameen, J. Hawkins, S. Lee, L. Li, A. Schwertschkow, G. Bauer, L. Lisowski, M. A. Kay, S. K. Kim, A. T. Lane, M. Wernig, A. E. Oro, Human COL7A1-corrected induced pluripotent stem cells for the treatment of recessive dystrophic epidermolysis bullosa. Sci. Transl. Med. 6, 264ra163 (2014). 10.1126/scitranslmed.3009540 PMC4428910 25429056

[47] S. J. Hedley, C. Layton, M. Heaton, K. H. Chakrabarty, R. A. Dawson, D. J. Gawkrodger, S. MacNeil, Fibroblasts play a regulatory role in the control of pigmentation in reconstructed human skin from skin types I and II. Pigment Cell Res. 15, 49–56 (2002). 11837456 10.1034/j.1600-0749.2002.00067.x

[48] R. R. Driskell, B. M. Lichtenberger, E. Hoste, K. Kretzschmar, B. D. Simons, M. Charalambous, S. R. Ferron, Y. Herault, G. Pavlovic, A. C. Ferguson-Smith, F. M. Watt, Distinct fibroblast lineages determine dermal architecture in skin development and repair. Nature 504, 277–281 (2013). 24336287 10.1038/nature12783 PMC3868929

[49] C. Philippeos, S. B. Telerman, B. Oules, A. O. Pisco, T. J. Shaw, R. Elgueta, G. Lombardi, R. R. Driskell, M. Soldin, M. D. Lynch, F. M. Watt, Spatial and single-cell transcriptional profiling identifies functionally distinct human dermal fibroblast subpopulations. J. Invest. Dermatol. 138, 811–825 (2018). 29391249 10.1016/j.jid.2018.01.016 PMC5869055

[50] D. G. Janson, G. Saintigny, A. van Adrichem, C. Mahe, A. El Ghalbzouri, Different gene expression patterns in human papillary and reticular fibroblasts. J. Invest. Dermatol. 132, 2565–2572 (2012). 22696053 10.1038/jid.2012.192

[51] L. Muhl, G. Genove, S. Leptidis, J. Liu, L. He, G. Mocci, Y. Sun, S. Gustafsson, B. Buyandelger, I. V. Chivukula, A. Segerstolpe, E. Raschperger, E. M. Hansson, J. L. M. Bjorkegren, X. R. Peng, M. Vanlandewijck, U. Lendahl, C. Betsholtz, Single-cell analysis uncovers fibroblast heterogeneity and criteria for fibroblast and mural cell identification and discrimination. Nat. Commun. 11, 3953 (2020). 32769974 10.1038/s41467-020-17740-1 PMC7414220

[52] V. S. LeBleu, E. G. Neilson, Origin and functional heterogeneity of fibroblasts. FASEB J. 34, 3519–3536 (2020). 32037627 10.1096/fj.201903188R

[53] A. M. Ascension, S. Fuertes-Alvarez, O. Ibanez-Sole, A. Izeta, M. J. Arauzo-Bravo, Human dermal fibroblast subpopulations are conserved across single-cell RNA sequencing studies. J. Invest Dermatol. 141, 1735–1744.e35 (2021). 33385399 10.1016/j.jid.2020.11.028

[54] A. Ni Annaidh, K. Bruyere, M. Destrade, M. D. Gilchrist, M. Ottenio, Characterization of the anisotropic mechanical properties of excised human skin. J. Mech. Behav. Biomed. Mater. 5, 139–148 (2012). 22100088 10.1016/j.jmbbm.2011.08.016

[55] P. Radermacher, P. Haouzi, A mouse is not a rat is not a man: Species-specific metabolic responses to sepsis—A nail in the coffin of murine models for critical care research? Intensive Care Med. Exp. 1, 26 (2013). 26266795 10.1186/2197-425X-1-7 PMC4796700

[56] S. R. Frost, A. Deodhar, G. J. Offer, A novel use for the biodegradable temporizing matrix. Eur. J. Plast. Surg. 45, 1015–1020 (2022). 35637749 10.1007/s00238-022-01964-z PMC9134145

[57] N. D. McKay, B. Robinson, R. Brodie, N. Rooke-Allen, Glucose transport and metabolism in cultured human skin fibroblasts. Biochim. Biophys. Acta 762, 198–204 (1983). 6830872 10.1016/0167-4889(83)90071-x

[58] E. A. Sander, K. A. Lynch, S. T. Boyce, Development of the mechanical properties of engineered skin substitutes after grafting to full-thickness wounds. J. Biomech. Eng. 136, 051008 (2014). 24356985 10.1115/1.4026290 PMC4023834

