# Supplementary material for: Engineering edgeless human skin with enhanced biomechanical properties
Source: Sci Adv. 2023 Jan 27;9(4):eade2514. doi: 10.1126/sciadv.ade2514 (PMC9882972; doi:10.1126/sciadv.ade2514)
Supplement: Supplementary file 1 — Figs. S1 to S20 [file sciadv.ade2514_sm.pdf]

Science Advances
AAAS

Supplementary Materials for

Engineering edgeless human skin with enhanced biomechanical properties

Alberto Pappalardo *et al.*

Corresponding author: Hasan Erbil Abaci, hea2113@cumc.columbia.edu

Sci. Adv. **9**, eade2514 (2023)
DOI: 10.1126/sciadv.ade2514

The PDF file includes:

Figs. S1 to S20
Legends for movies S1 to S3

Other Supplementary Material for this manuscript includes the following:

Movies S1 to S3

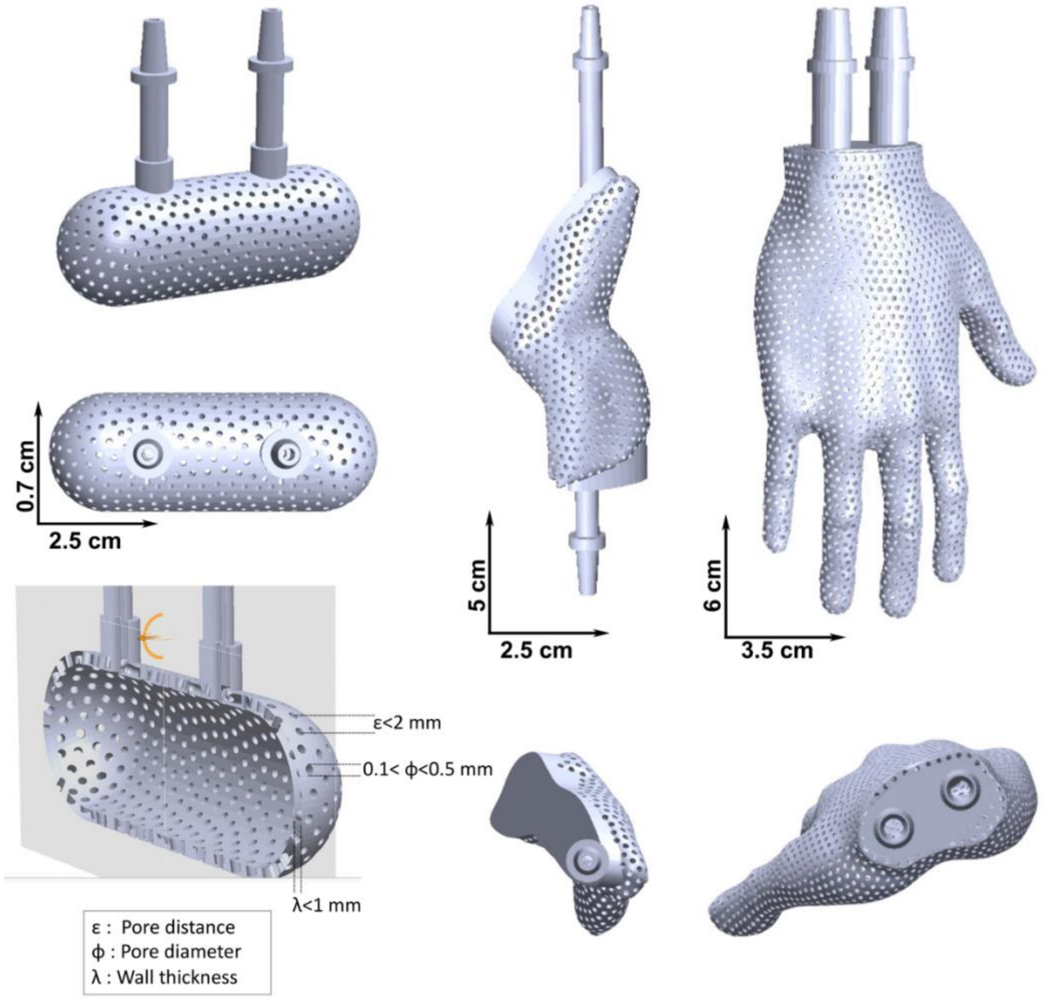

Figure S1. CAD models of the scaffolds with specifics. The total height and width for all geometries are given. All scaffolds share the surface design with a thickness of approximately 0.7 mm and pores of 0.5 mm in diameter distanced 2 mm from one another.

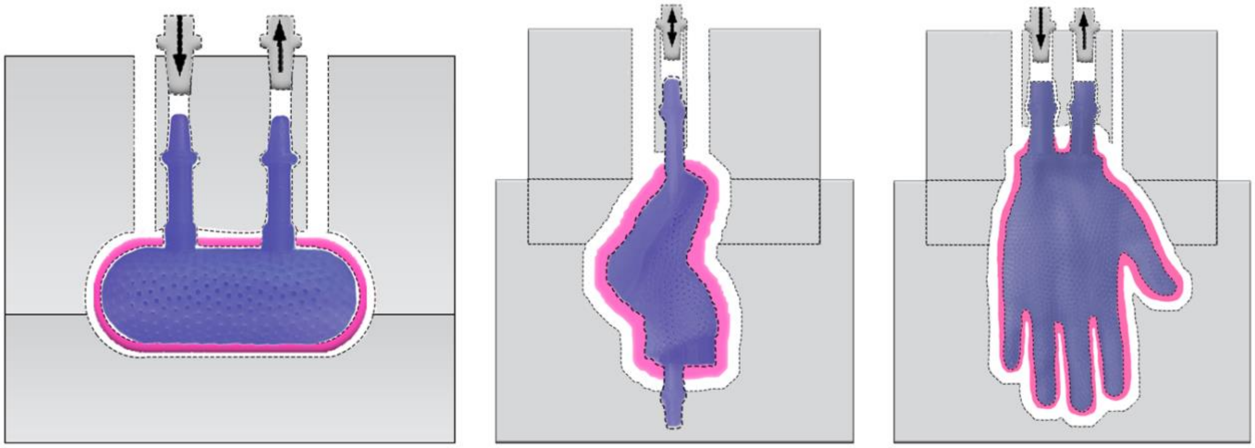

Figure S2. Illustration showing the three scaffolds inside the PDMS molds. From left to right, cylinder, mouse hindlimb, and human hand.

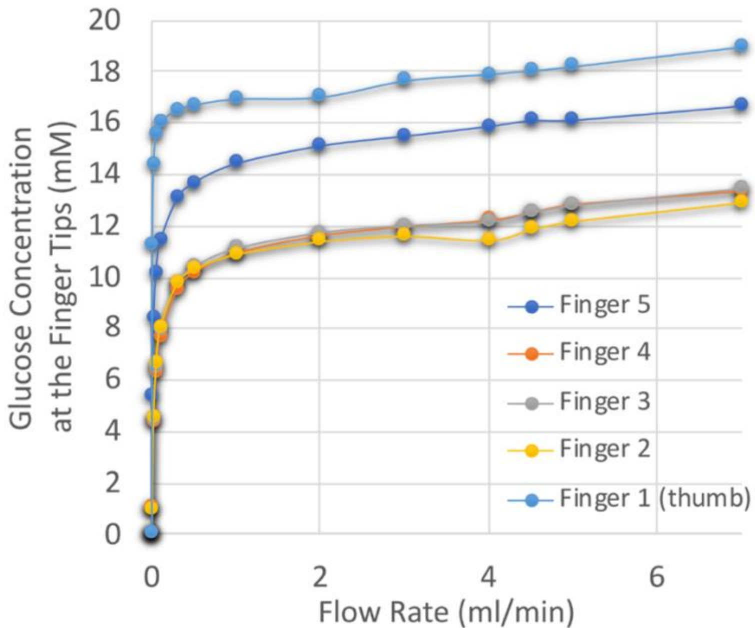

Figure S3. COMSOL simulation of the glucose concentration at five finger tips for various medium flow rates. Following the analysis, a perfusion rate of 5 ml min^{-1} was chosen for the culture of the hand constructs.

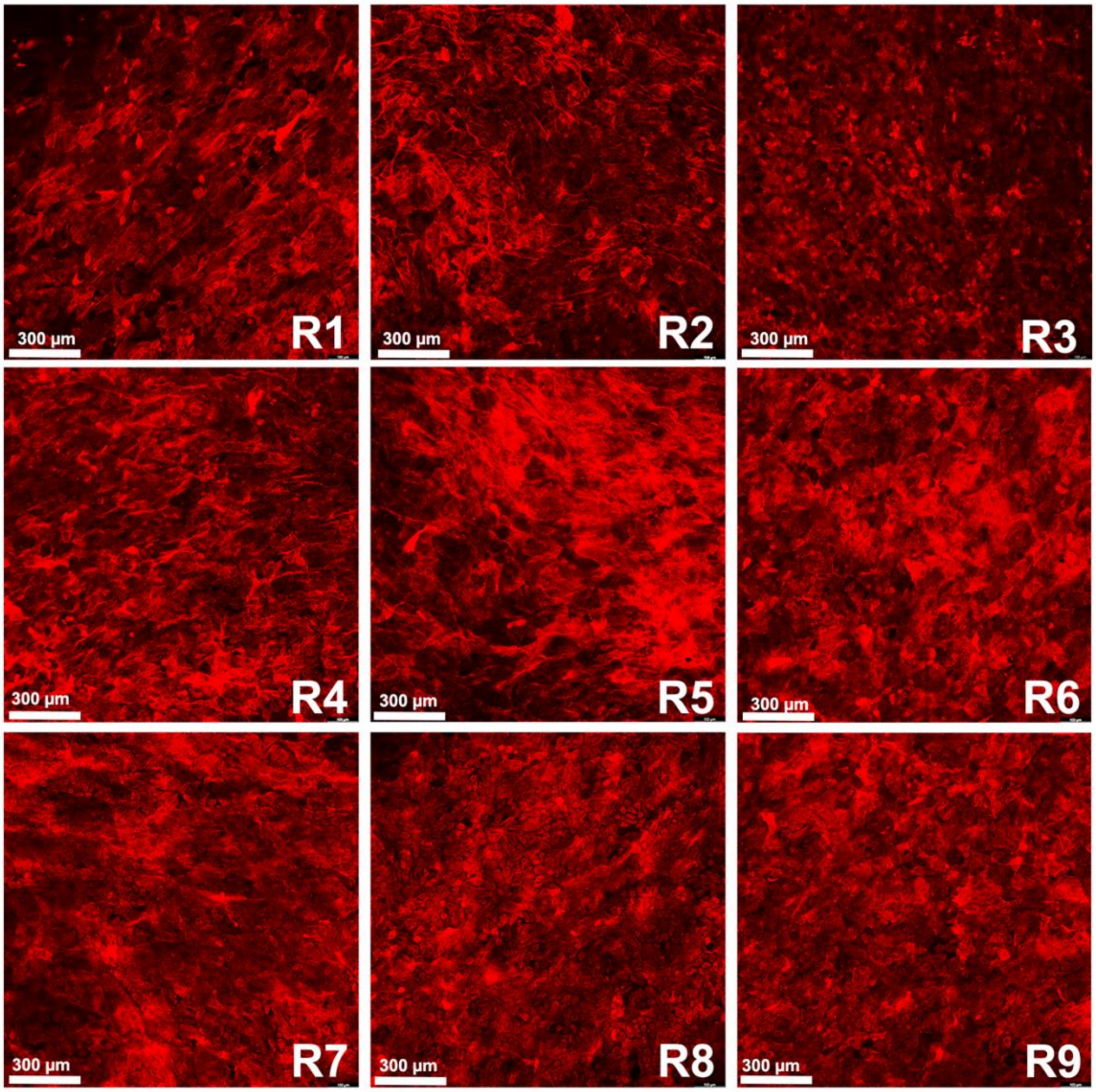

Figure S4. Skin glove epideral coverage. Epidermal coverage in nine representative regions (R) of the skin glove including finger tips, knuckles, palm and wrist**.** Scale bars: 300 µm.

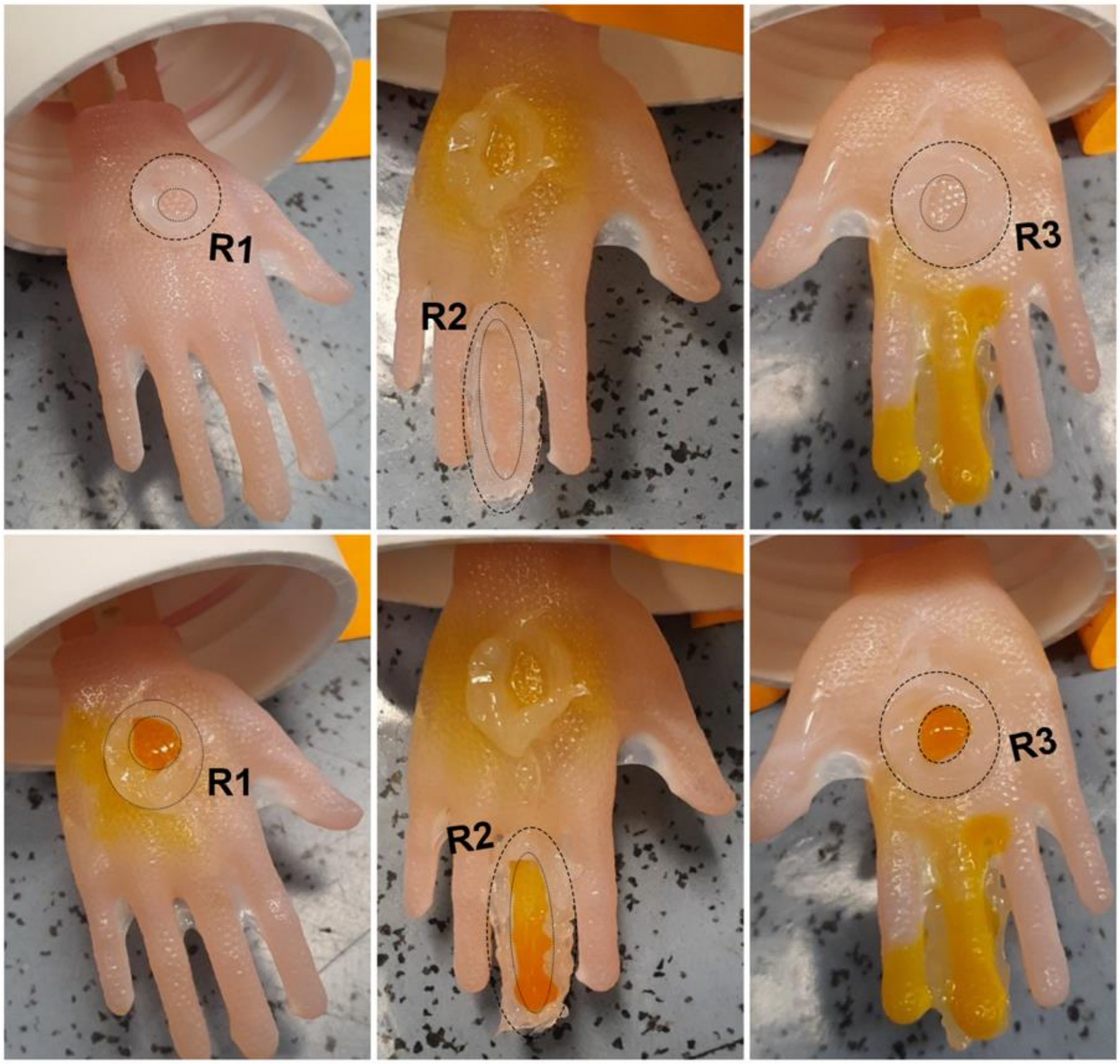

Figure S5. Lucifer Yellow permeability test. Three different regions were sequentially isolated with paraffin gel before the application of the fluorescent dye.

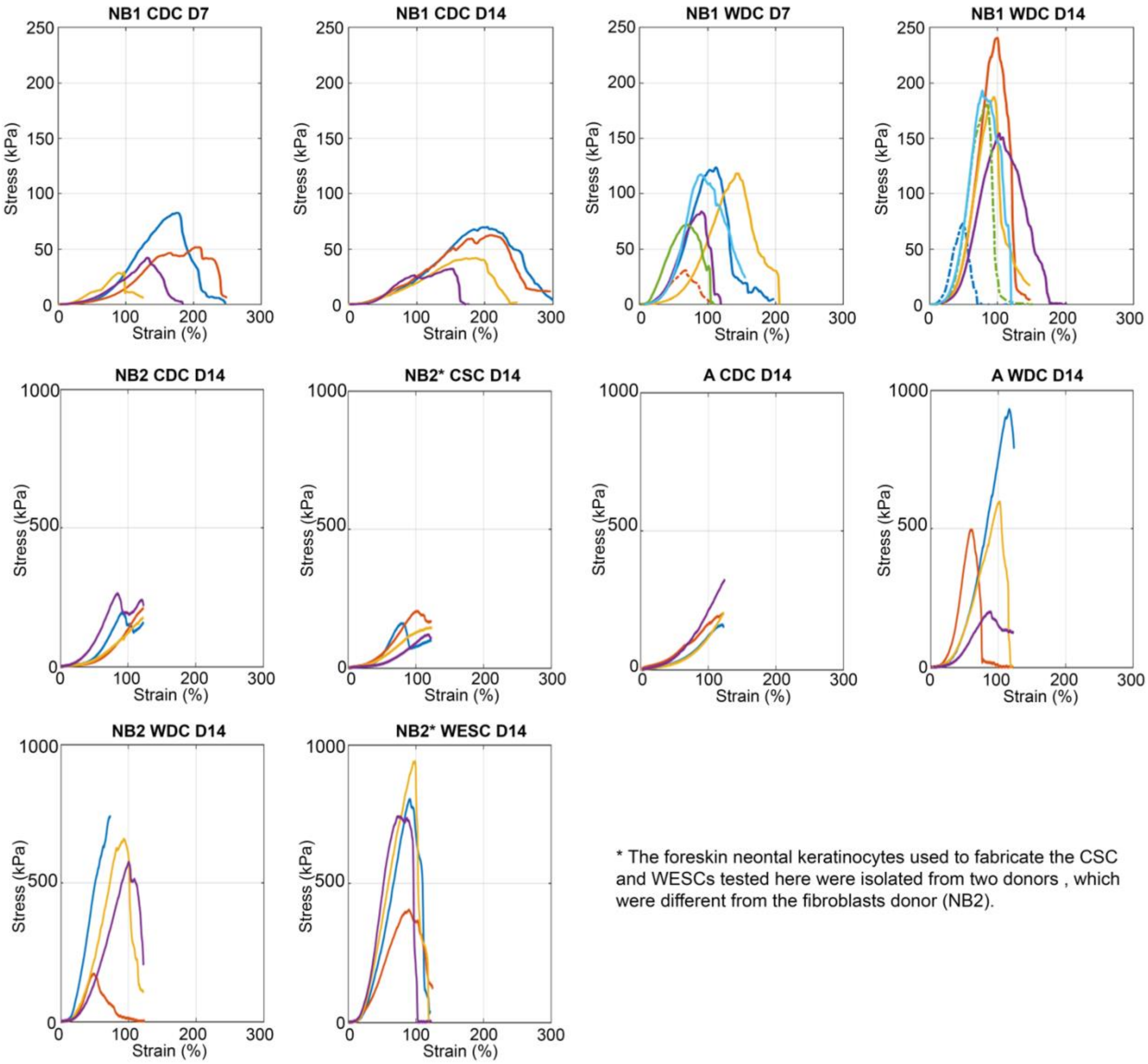

Figure S6. Stress-strain curves for WDCs and CDCs for all conditions. The CDCs seemed capable of a greater strain before rupturing compared to the WDCs, but no significant difference emerged from the statistical analysis. Each colored line represents one sample, while dashed lines represent samples that slipped off the grip during the test.

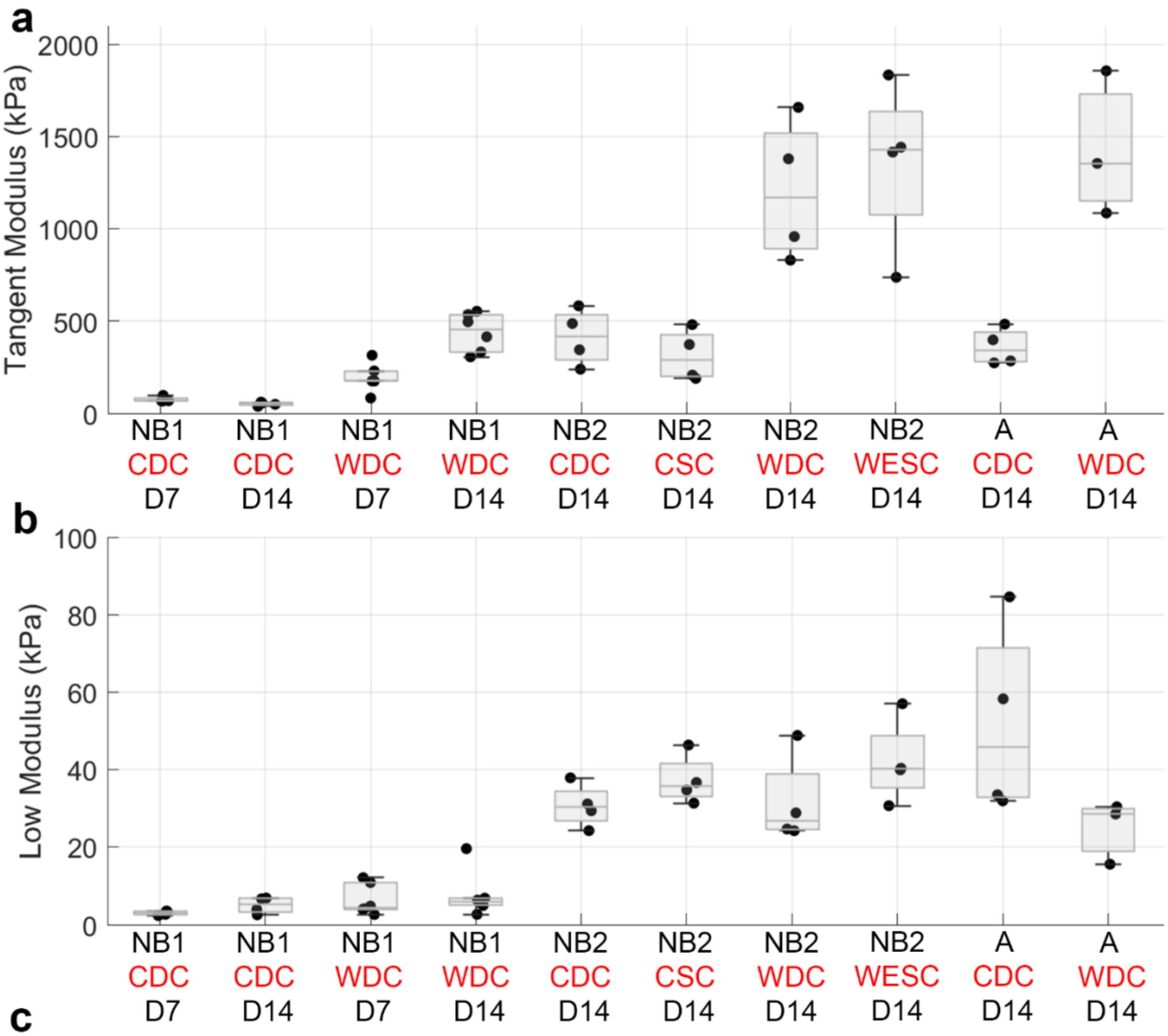

c

SAMPLE	NB1 CDC D7	NB1 CDC D14	NB1 WDC D7	NB1 WDC D14	NB2 CDC D14	NB2 CSC D14	NB2 WDC D14	NB2 WESC D14	A CDC D14	A WDC D14	
NB1 CDC D7		ns	ns	ns	*	***	**	***	****	ns	low modulus
NB1 CDC D14	ns		ns	ns	*	**	*	***	****	ns	
NB1 WDC D7	ns	ns		ns	*	**	*	***	****	ns	
NB1 WDC D14	****	****	***		*	**	*	***	****	ns	
NB2 CDC D14	ns	ns	ns	ns		ns	ns	ns	ns	ns	
NB2 CSC D14	ns	ns	ns	ns	ns		ns	ns	ns	ns	
NB2 WDC D14	****	****	****	***	***	****		ns	*	ns	
NB2 WESC D14	****	****	****	****	****	****	ns		ns	ns	
A CDC D14	ns	ns	ns	ns	ns	ns	***	****		ns	
A WDC D14	****	****	****	****	****	****	ns	ns	****		
	tangent modulus										

Figure S7. Graphs showing the absolute level of force achieved for the tangent modulus and low modulus during the uniaxial stretch test. a) Tangent modulus. b) Low modulus c) statistical analysis (* = p<0.05, ** = p<0.01, *** = p<0.001, **** = p<0.0001 .

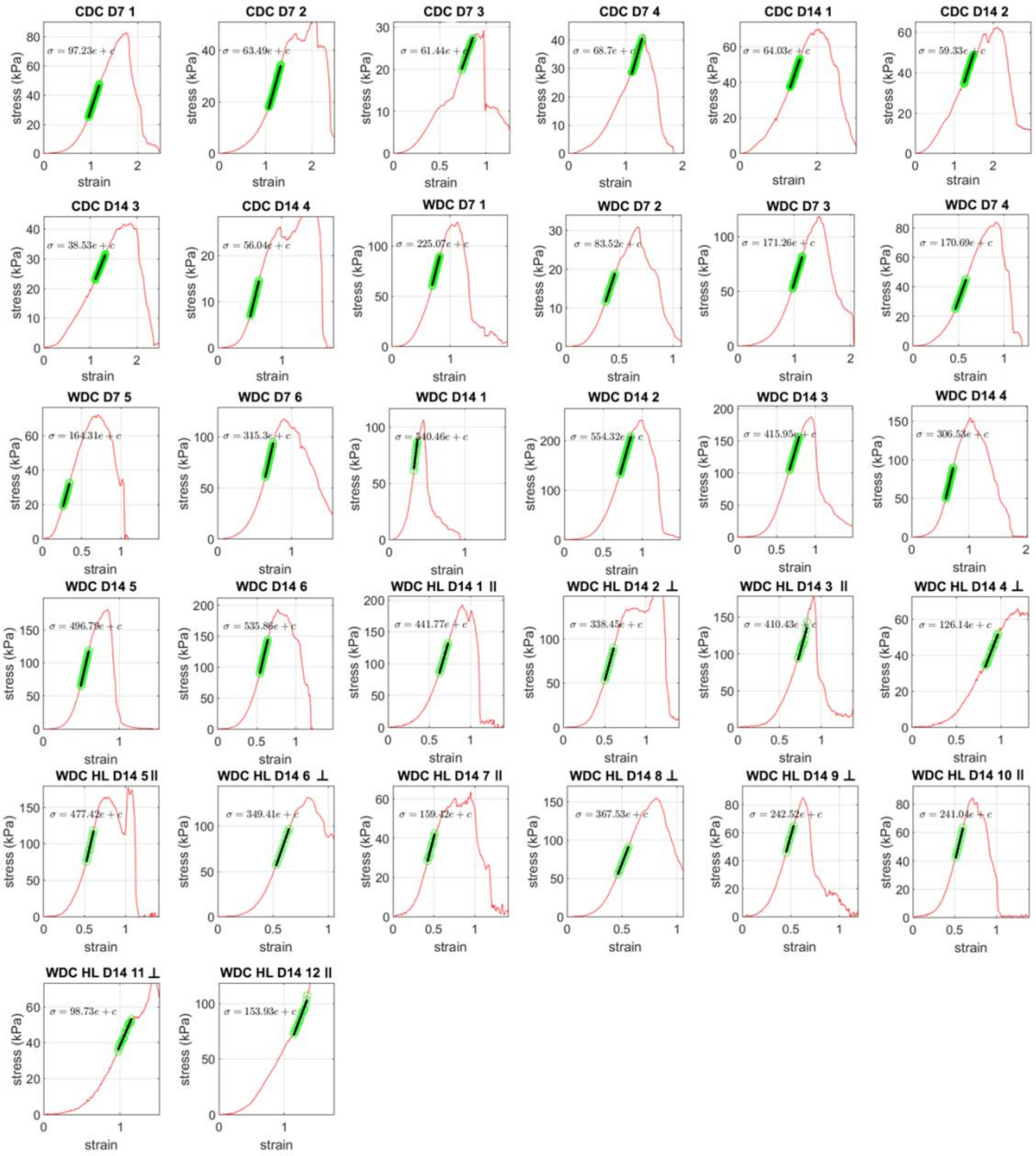

Figure S8. Uniaxial stretch test stress/strain single plots for all constructs generated with NB1 fibroblasts. a) Graphs plotting the stress for increasing levels of strain during the test. These plots were employed to calculate the tangent modulus (green) and the low modulus. The hindlimb (HL) samples were stretched either in parallel (II) or perpendicularly (⊥) to the direction of the collagen fibers.

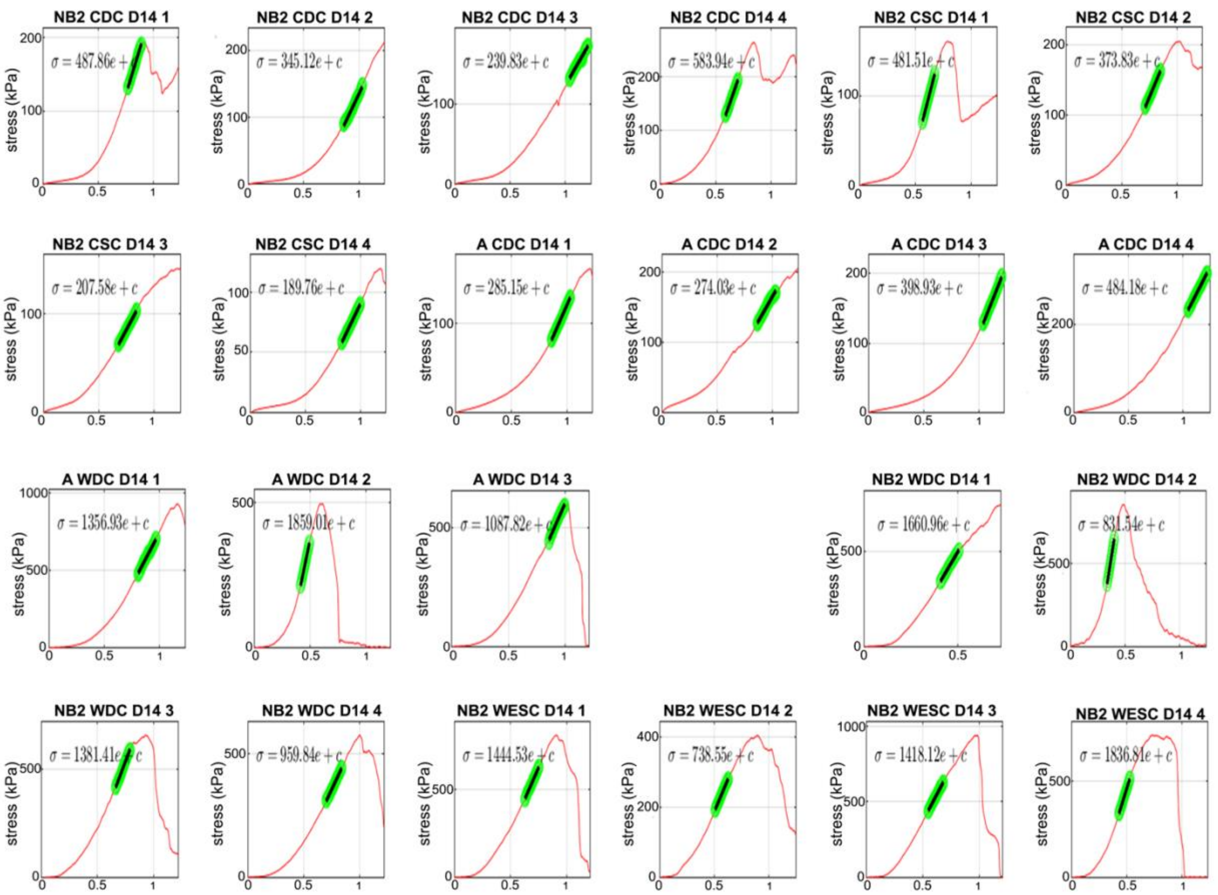

Figure S9. Uniaxial stretch test stress/strain single plots for constructs generated with fibroblasts from donors NB2 and A. a) Graphs plotting the stress for increasing levels of strain during the test. These plots were employed to calculate the tangent modulus (green) and the low modulus.

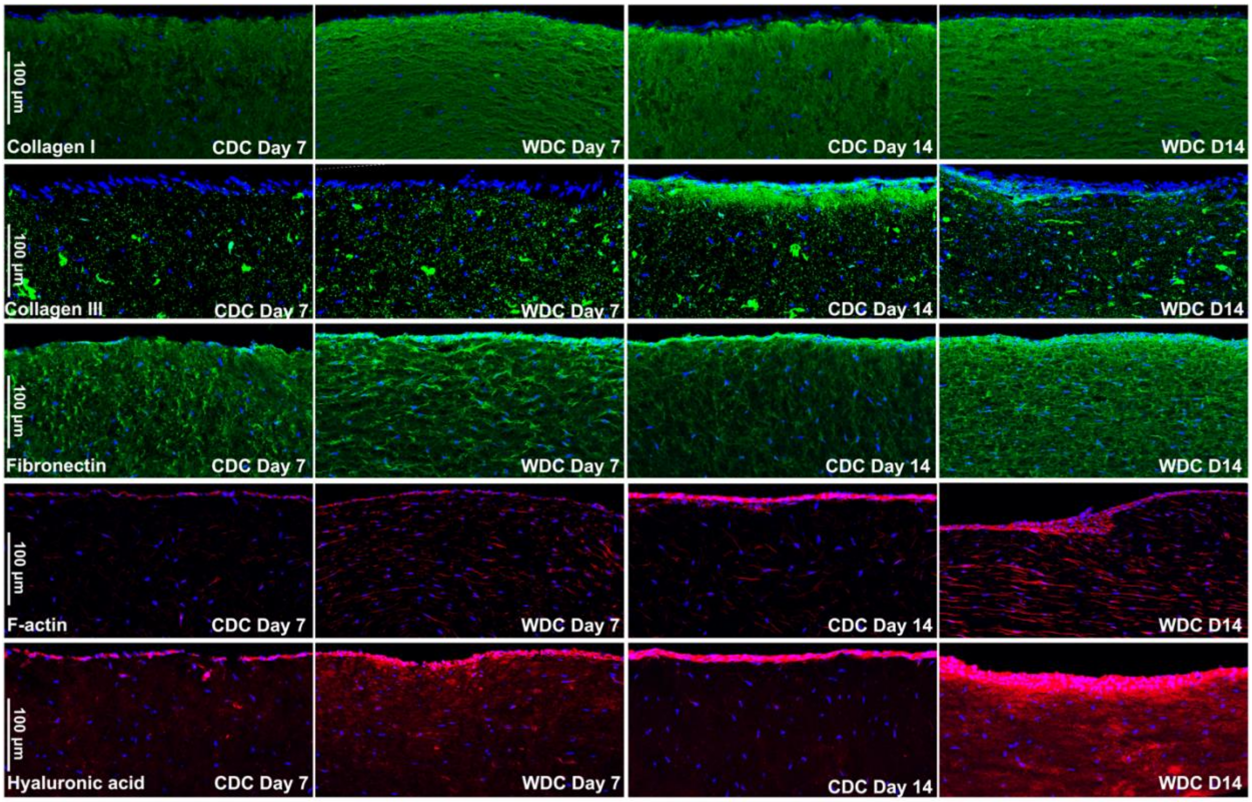

Figure S10. IF images of principal ECM proteins of the dermis from CDCs and WDCs. Scale bars: 100 µm.

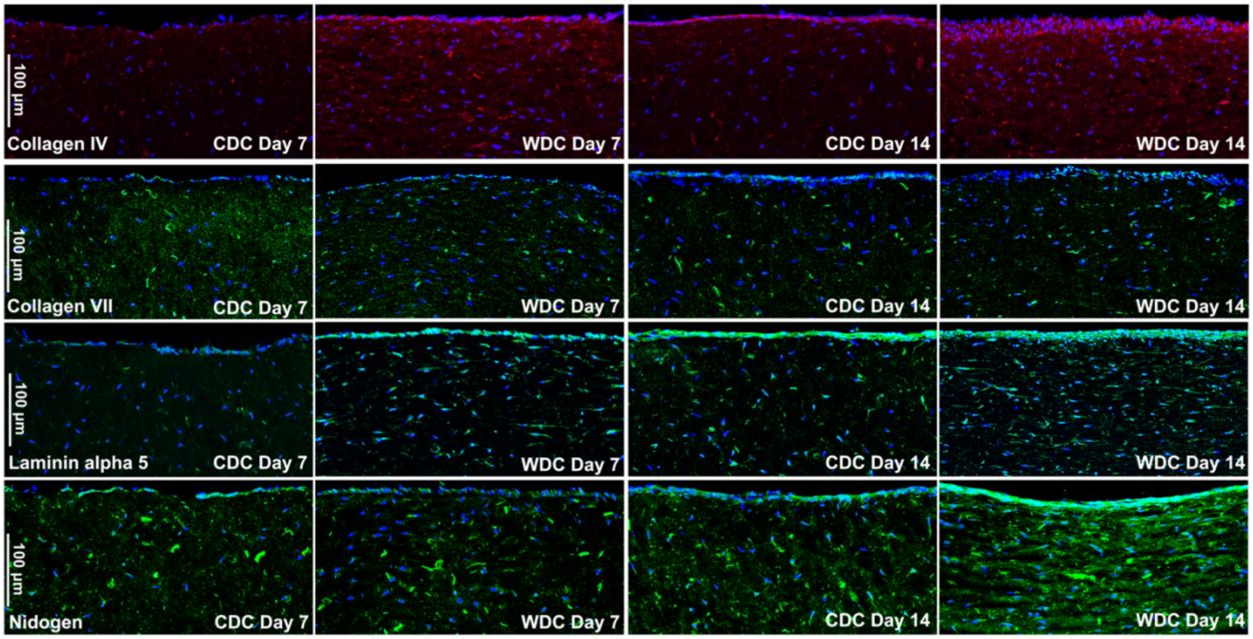

Figure S11. Extra IF images of principal BM proteins. The WDCs show an increased accumulation of BM proteins throughout the construct, but especially on their surface. Scale bars: 100 µm.

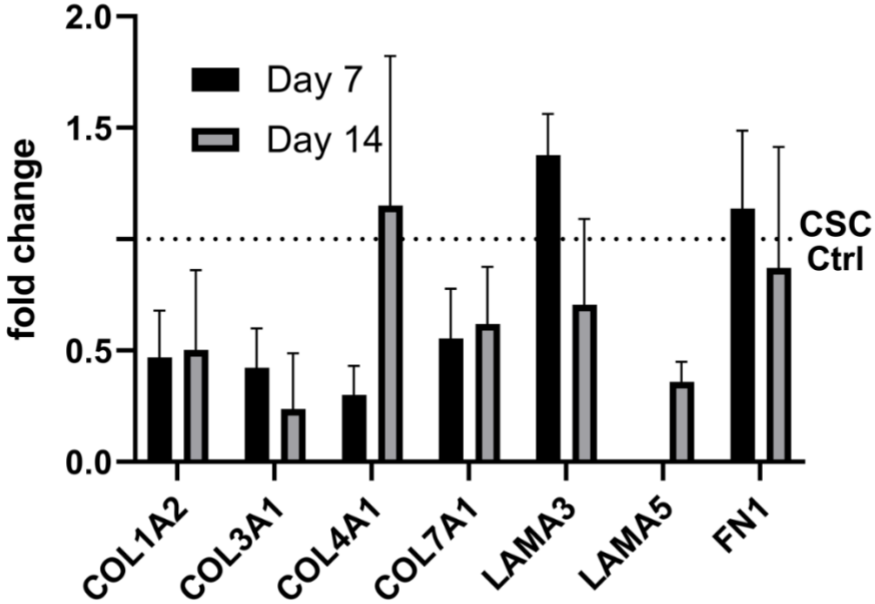

Figure S12. qPCR analysis of relevant ECM genes expression. No significant differences were found between CDCs and WDCs.

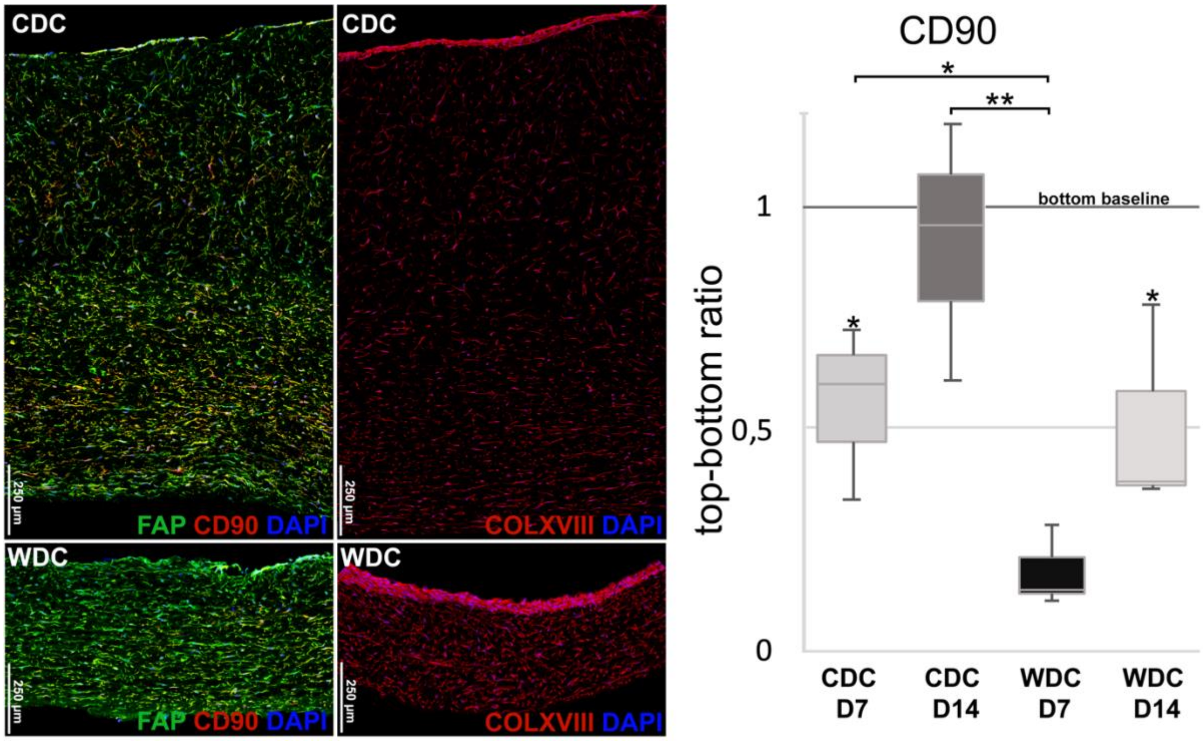

Figure S13. Characterization of papillary and reticular markers through IF. The expression of FAP, CD90 (left panel) and collagen XVIII (central panel) were assessed with IF for CDC and WDC. While FAP and collagen XVIII result homogenous throughout, when the fluorescence intensity is normalized by cell number, the expression of CD90 (right graph) resulted significantly decreased in the upper 15% of the dermal thickness at day 7 for both CDC and WDC, while at day 14 only WDC remained significantly downregulated. Scale bars: 250 µm.

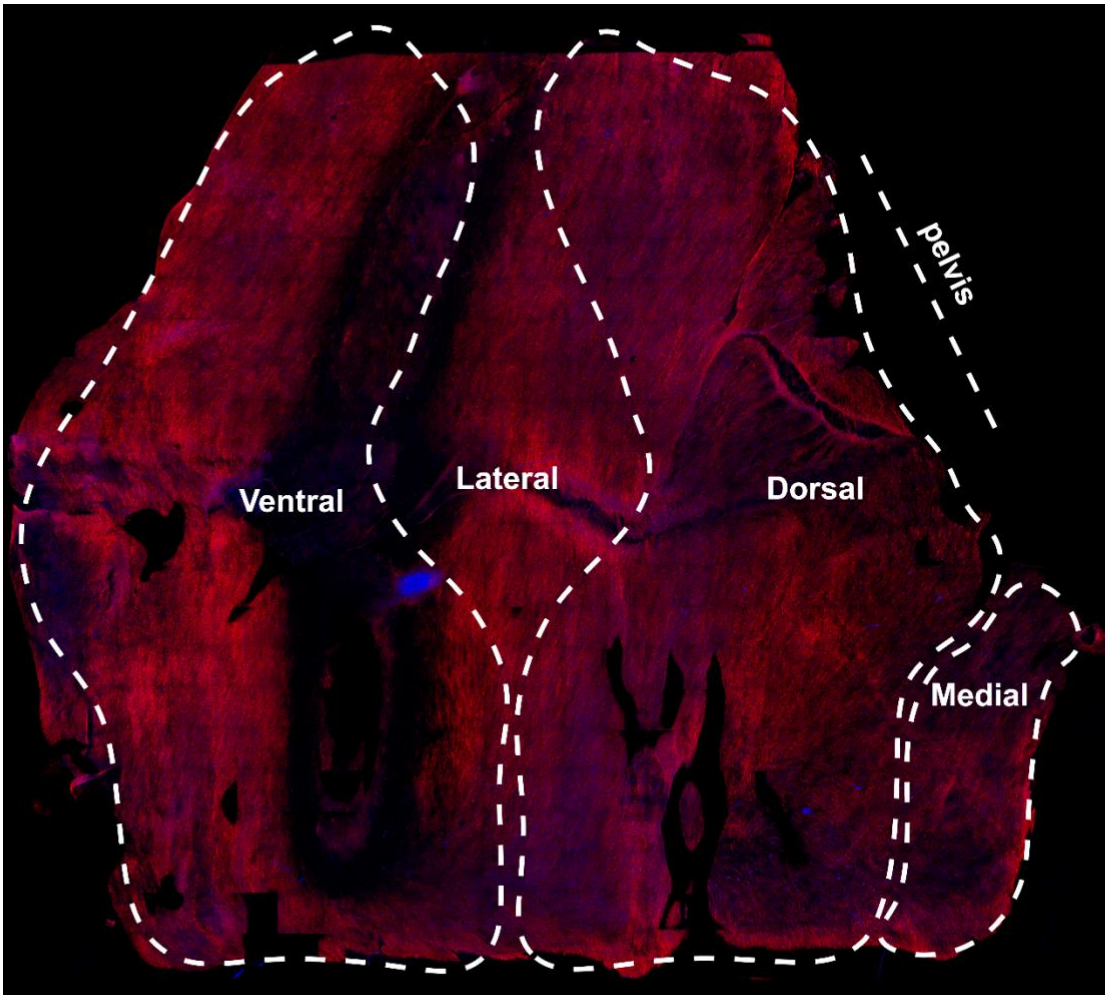

Figure S14. F-actin map of the WHCs. Dermis-only hindlimb constructs were fluorescently labeled for F-actin as a whole-mount tissue. A 3D scan for the whole surface area is given for the corresponding regions of the hindlimb.

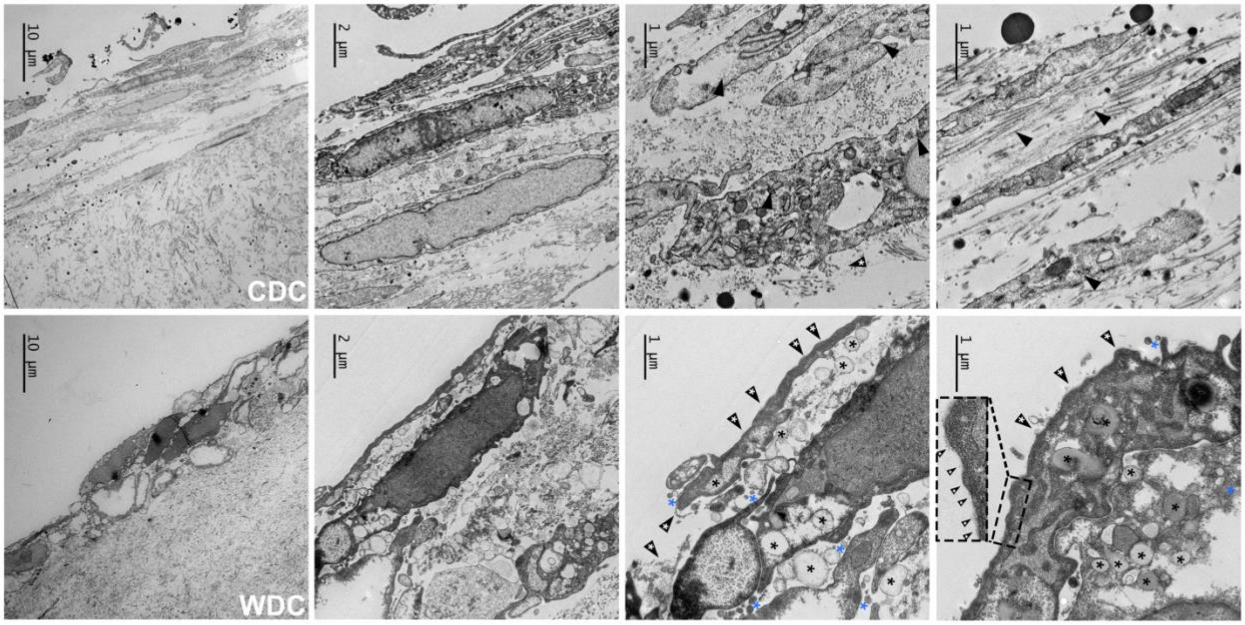

Figure S15. Images comparing CDC and WDC at high magnification acquired through TEM. The top row shows the CDC with flattened fibroblasts parallel to the surface and the presence of thick collagen fibers, likely collagen I or III (black arrowheads), as opposed to fibrillar basement membrane proteins such as collagen IV and laminin. On the contrary the WDC in the bottom row shows larger cells, containing a high number of secretion vesicles rich of thin fibrillar material (black stars) and were surrounded by numerous exosomes (blue stars). On the surface WDCs present a thin layer of fibrillae of about 25 - 40 nm (black arrowheads with white stars), which is missing in CDCs. Scale bars: from the left 10 µm (first image column), 2 µm (second column), 1 µm (third and fourth image columns).

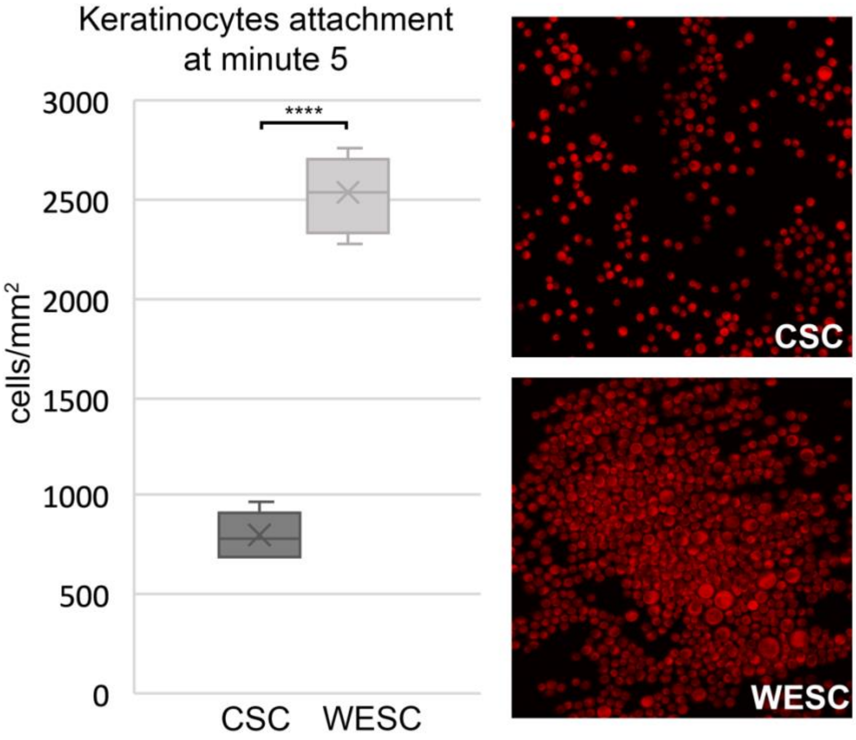

Figure S16. Keratinocytes attachment/retention assay. WESCs (bottom right) presented a 4-fold higher KCs density compared to CSCs.

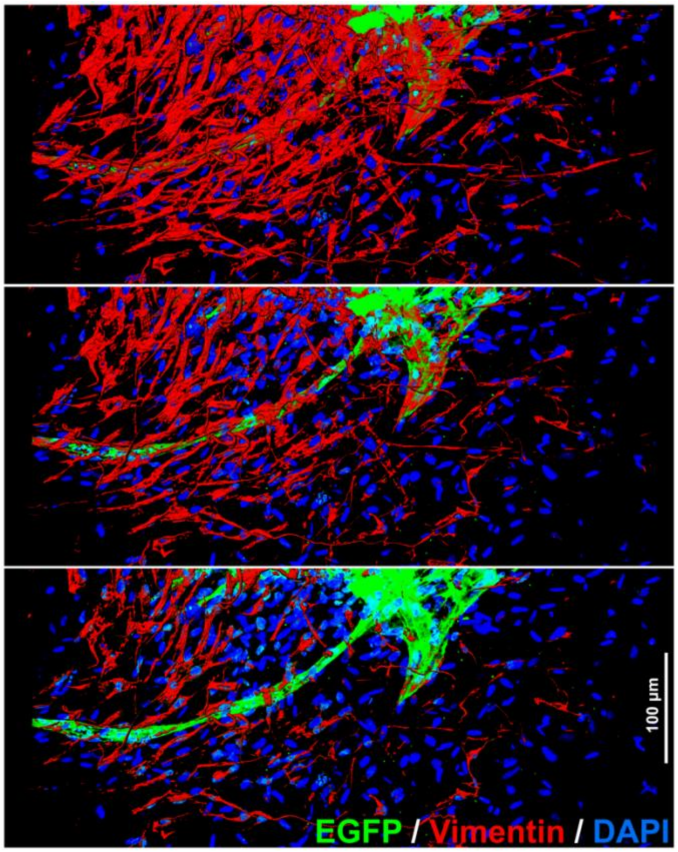

Figure S17. 3D rendering with volumetric reconstruction of a Z stack series showing a capillary sprout and its relation with the surrounding FBs. The same image is shown at progressively decreased level of exposure of Vimentin (red) to showcase the direct interaction between FBs and HDBECs. The capillary is wrapped by the FBs and guided through the ECM. Scalebar: 100 µm.

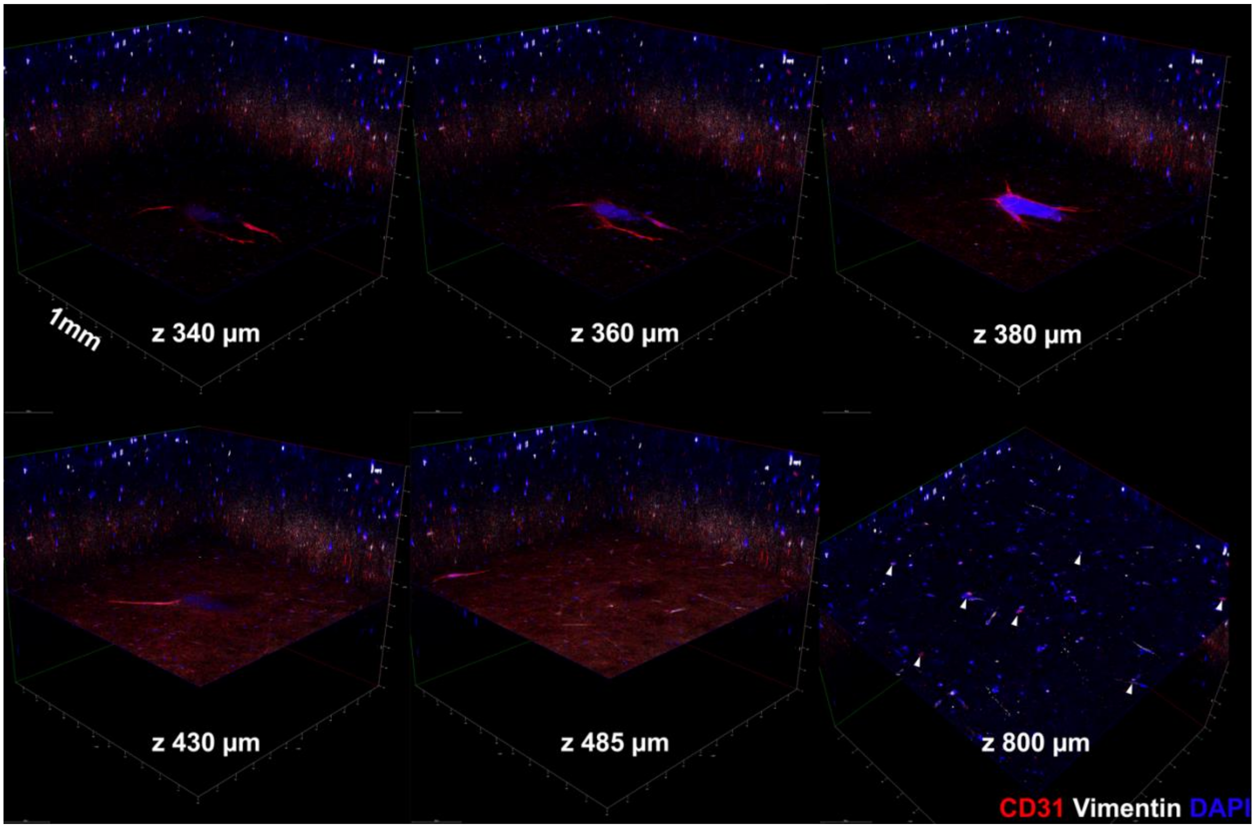

Figure S18. Z stack series showing the vascularization of a WDC after eighteen days of culture in vitro. The EC aggregate migrated within the construct for about 350 µm and sprouted for additional 130 µm towards the surface. Single endothelial cells are visible up to 800 µm in the dermal thickness. The construct was stained as a wholemount with CD31 (red) and DAPI (blue). Total axis length x,y,z = 1000 µm.

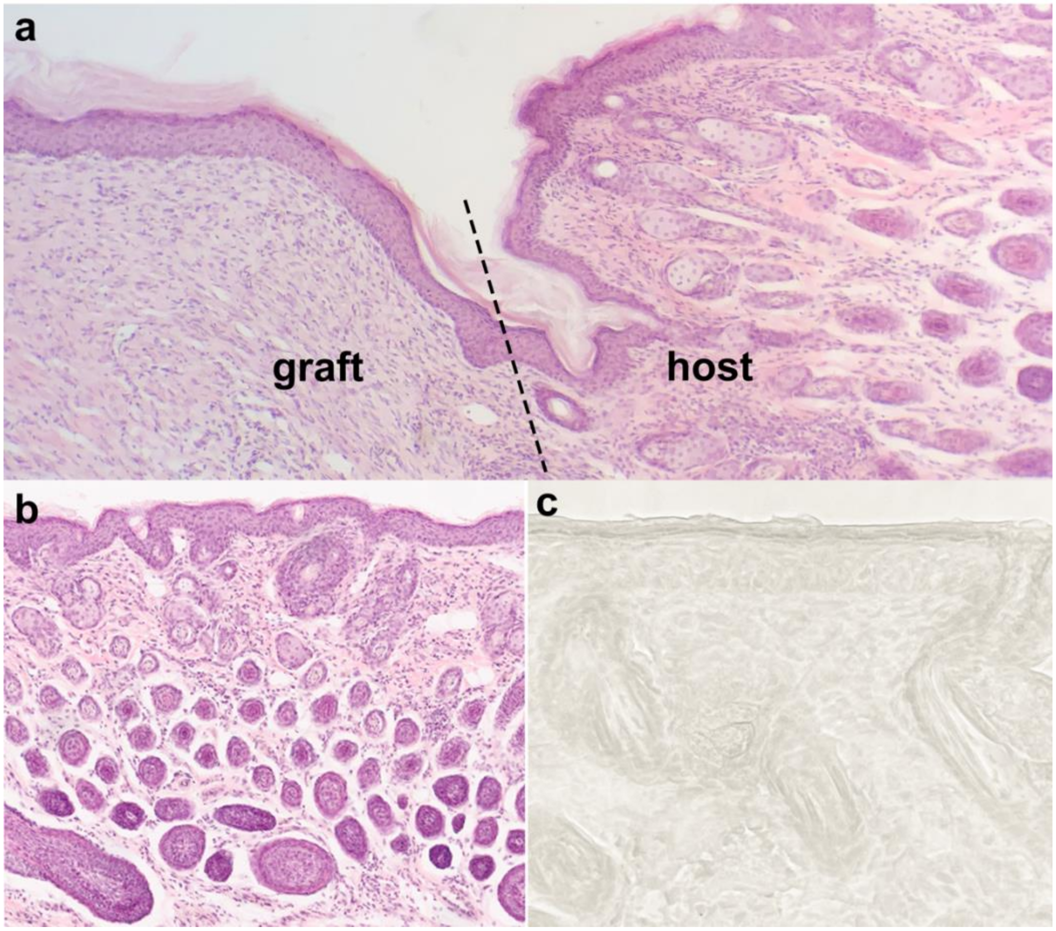

Figure S19. Normal mouse skin histology and IHC. a) H&E of graft-host skin interface. **b)** H&E of normal mouse skin outside the wound region. H&E of mouse-graft interface. **c)** Control IHC staining for human involucrin showing normal mouse skin which results negative.

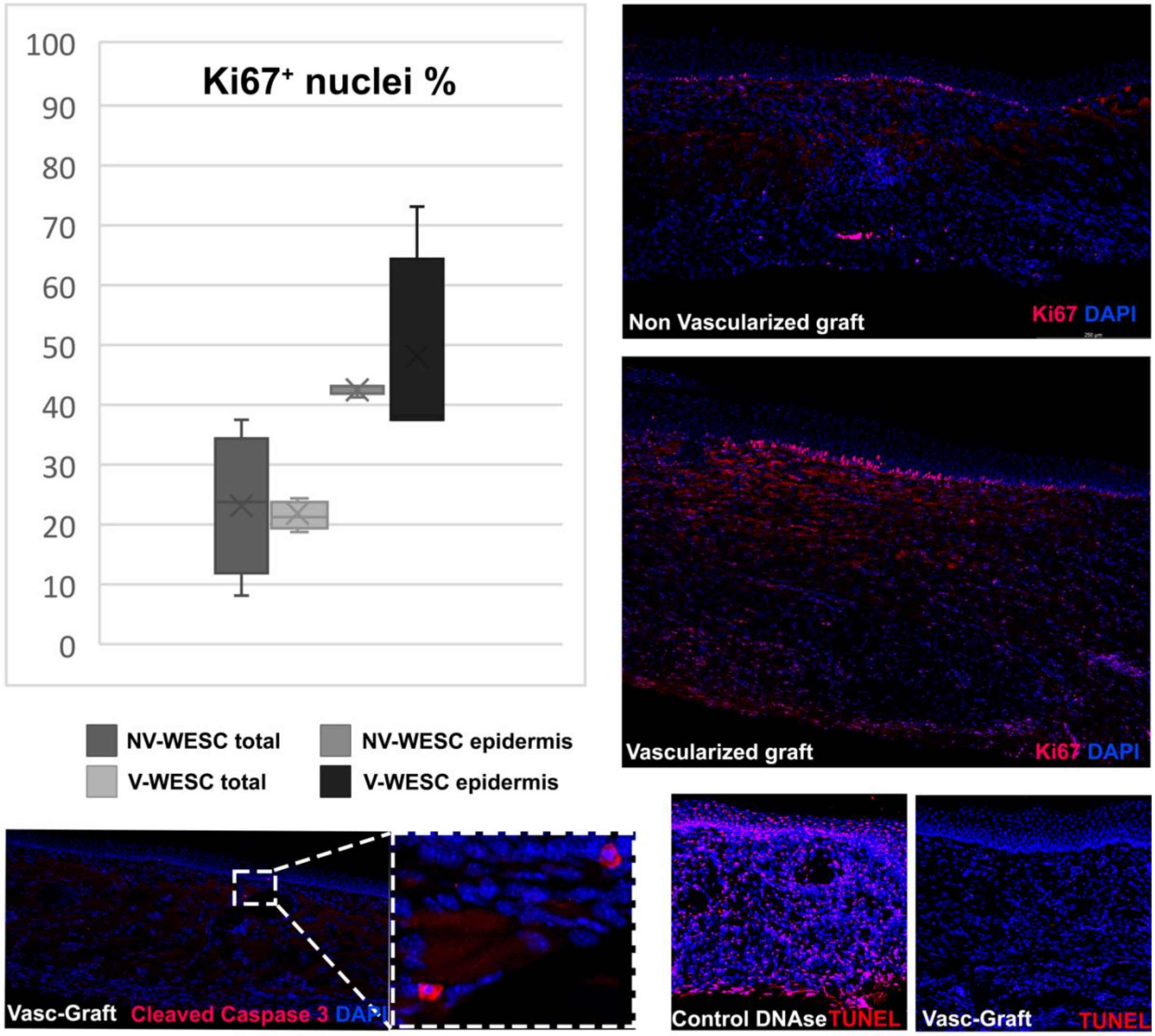

Figure S20. Comparison of cell proliferation and apoptosis in vascularized and non-vascularized grafts. Cell proliferation was comparable in vascularized and non-vascularized grafts, and the basal layer of the epidermis showed a higher rate of proliferation with a rough average 45% positive nuclei. The apoptosis marker Cleaved Caspase 3 (bottom left) and the DNA fragmentation assay TUNEL (bottom right) were uniformly minimal or undetectable in the two conditions, demonstrating the integration of the grafts.

Movie S1 (separate file). After maturation, the hand glove can be easily removed from its scaffold and used for downstream applications.

Movie S2 (separate file). The transplantation of the hindlimb graft it's a very fast and seamless procedure requiring only a few stitches, as opposed to traditional flat skin patches.

Movie S3 (separate file). After four weeks from the grafting surgery the mouse completely recovered and show no loss of function in the transplanted limb.
